# Post-translational modification analysis of *Saccharomyces cerevisiae* histone methylation enzymes reveals phosphorylation sites of regulatory potential

**DOI:** 10.1074/jbc.RA120.015995

**Published:** 2020-12-20

**Authors:** Ryan J. Separovich, Mandy W.M. Wong, Tyler R. Chapman, Eve Slavich, Joshua J. Hamey, Marc R. Wilkins

**Affiliations:** 1 School of Biotechnology and Biomolecular Sciences, University of New South Wales, Sydney, New South Wales, Australia; 2 Stats Central, Mark Wainwright Analytical Centre, University of New South Wales, Sydney, New South Wales, Australia

**Keywords:** chromatin, histone methylation, epigenetics, post-translational modification, phosphorylation, kinase, mass spectrometry, *Saccharomyces cerevisiae*, methyltransferase, demethylase, COMPASS, complex of proteins associated with Set1, DMase, demethylase, Dot1, disruptor of telomeric silencing 1, EThcD, electron transfer higher-energy collisional dissociation, FDR, false discovery rate, Gis1, GIg1-2 suppressor 1, HCD, higher-energy collisional dissociation, JmjC, Jumonji C, JmjN, Jumonji N, MTase, methyltransferase, *m/z*, mass to charge ratio, mmu, milli mass unit, PHD, plant homeodomain, ppm, parts per million, PSM, peptide-spectrum match, PTM, post-translational modification, Rph1, regulator of Phr1, rpm, revolutions per minute, SC-ura, synthetic complete medium lacking uracil, SET, Su(var)3-9, Enhancer of Zeste, Trithorax, SRI, Set2 Rbp1 interacting, SUMO, small ubiquitin-like modifier, XIC, extracted ion chromatogram

## Abstract

Histone methylation is central to the regulation of eukaryotic transcription. In *Saccharomyces cerevisiae*, it is controlled by a system of four methyltransferases (Set1p, Set2p, Set5p, and Dot1p) and four demethylases (Jhd1p, Jhd2p, Rph1p, and Gis1p). While the histone targets for these enzymes are well characterized, the connection of the enzymes with the intracellular signaling network and thus their regulation is poorly understood; this also applies to all other eukaryotes. Here we report the detailed characterization of the eight *S. cerevisiae* enzymes and show that they carry a total of 75 phosphorylation sites, 92 acetylation sites, and two ubiquitination sites. All enzymes are subject to phosphorylation, although demethylases Jhd1p and Jhd2p contained one and five sites respectively, whereas other enzymes carried 14 to 36 sites. Phosphorylation was absent or underrepresented on catalytic and other domains but strongly enriched for regions of disorder on methyltransferases, suggesting a role in the modulation of protein–protein interactions. Through mutagenesis studies, we show that phosphosites within the acidic and disordered N-terminus of Set2p affect H3K36 methylation levels *in vivo*, illustrating the functional importance of such sites. While most kinases upstream of the yeast histone methylation enzymes remain unknown, we model the possible connections between the cellular signaling network and the histone-based gene regulatory system and propose an integrated regulatory structure. Our results provide a foundation for future, detailed exploration of the role of specific kinases and phosphosites in the regulation of histone methylation.

Histone methylation is a widespread and dynamic post-translational modification (PTM) that is central to the regulation of eukaryotic transcription (1). It regulates gene expression by recruiting transcriptional cofactors that harbor domains to specifically recognize methylated lysine or arginine residues (2). Importantly, different histone methylation marks display unique chromosomal distributions in and around genes and carry out distinct biological roles (3, 4). The functional importance of histone methylation is underscored by the many diseases associated with its dysregulation, including cancers, neurodevelopmental defects, and blood cell disorders (5, 6).

The histone methylation system is highly conserved among eukaryotes; from the methylation sites themselves to the enzymatic machinery responsible for their regulation. The budding yeast *Saccharomyces cerevisiae* retains the transcriptionally activating lysine methylation sites on H3K4, H3K36, and H3K79 (7), in addition to H4K5, H4K8, and H4K12 monomethylation sites (8). Crucially, the genomic distribution of methylation sites changes during yeast cellular growth and in response to exogenous perturbation, bringing about widespread transcriptional reprogramming (9). Histone lysine methylation in *S. cerevisiae* is controlled by four methyltransferase (MTase) and four demethylase (DMase) enzymes. The SET (Su(var)3-9, Enhancer of Zeste, Trithorax) domain MTases Set1p, Set2p, and Set5p methylate histone proteins on their N-terminal tails (10, 11), while Dot1p contains a seven-β-strand catalytic domain and methylates lysine 79 on the globular core of histone H3 (12, 13). Demethylation of histone lysine residues is catalyzed by the Jumonji domain-containing DMases Jhd1p and Jhd2p, as well as the paralogous enzymes Rph1p and Gis1p, which have contentious overlapping H3K36 DMase activity *in vivo* (14, 15, 16). Although the yeast histone methylation network is substantially simplified in comparison with its human counterpart, which comprises 35 histone MTases and 23 DMases (6, 17, 18, 19), almost all enzymes in *S. cerevisiae* have a mammalian ortholog, with the exception of Set5p.

Given their fundamental role in eukaryotic transcription, there is a pressing need to determine how histone MTase and DMase enzymes are regulated. Several foundational studies in human have begun to highlight the diverse effects of different PTMs on histone MTase and DMase function (20, 21, 22, 23), and phosphorylation in particular is emerging as a major regulator of mammalian histone methylation (24). Kinases from a range of human signaling pathways transmit information to histone MTases and DMases to fine-tune their catalytic activity (25), protein–protein interactions (26), chromatin binding (27), and stability (28), in response to intracellular and extracellular cues. Despite modest progress in deciphering the regulation of histone methylation in the human cell, there remains a paucity of understanding of how this process is controlled in yeast. Just a handful of targeted studies in *S. cerevisiae* have elucidated the function of PTM sites on histone MTases and DMases (15, 29, 30) or determined the upstream modifying enzymes responsible for their deposition (16, 31, 32). The regulatory roles of all other PTMs on yeast histone MTase and DMase function remain unknown, and as such, the connections between the intracellular protein interaction network and the histone methylation system are poorly understood. This considerable and surprising knowledge gap necessitates further functional analyses in *S. cerevisiae* to clarify the regulatory mechanisms underpinning histone methylation and thereby determine if certain types of post-translational regulation are conserved among eukaryotes or unique to yeast.

A comprehensive catalog of modification types and sites on an enzyme is requisite to understanding its post-translational regulation. Although several enrichment-based proteomic studies have identified many PTMs on histone MTases and DMases in *S. cerevisiae* (33, 34, 35, 36, 37, 38), there has been no systematic characterization of any specific methylation enzymes carried out to date. To this end, we homologously overexpressed and purified all yeast histone MTases and DMases and performed a combinatorial mass spectrometric pipeline (39) to comprehensively identify high-confidence PTM sites of various types. Our multi-protease, multi-fragmentation method approach achieved near-complete protein sequence coverage, allowing us to identify phosphorylation sites on all enzymes that were analyzed. To investigate the potential regulatory role of phosphorylation, we contextualized phosphosites within several important sequence and structural features of MTase and DMase enzymes, including protein domains, intrinsically disordered regions, and molecular interaction interfaces. Using mutagenesis studies, we experimentally confirmed the functional role of a phosphorylation cluster identified within an acidic and intrinsically disordered N-terminal region of the H3K36 MTase, Set2p. Significantly, our phosphosite mapping allowed us to model possible connections to the intracellular signaling network and thereby construct a draft regulatory network of histone methylation in yeast; the first of its kind for any epigenetic modification in eukaryotes. In addition to phosphorylation, we also identified acetylation and ubiquitination sites on certain enzymes but found no evidence for protein methylation, ADP-ribosylation, SUMOylation, or crotonylation. The draft phosphoregulatory network presented herein will be a valuable resource for targeted functional studies into the post-translational regulation of histone methylation.

## Results

### Comprehensive discovery of post-translational modifications using orthogonal mass spectrometric techniques

To systematically identify the PTMs on all yeast histone MTases and DMases, we employed enzyme purification and a combinatorial mass spectrometric approach. This involved four proteolytic digestions (trypsin, LysargiNase, Asp-N, and chymotrypsin) and two mass spectrometry fragmentation methods (higher-energy collisional dissociation (HCD) and electron transfer/HCD (EThcD)), applied to homologously overexpressed and purified versions of enzymes. Seven of all eight of the histone methylation proteins could be analyzed; Set1p was refractory to overexpression in *S. cerevisiae*. Note however that some PTMs for Set1p could be collated from literature sources.

**Figure 1:**
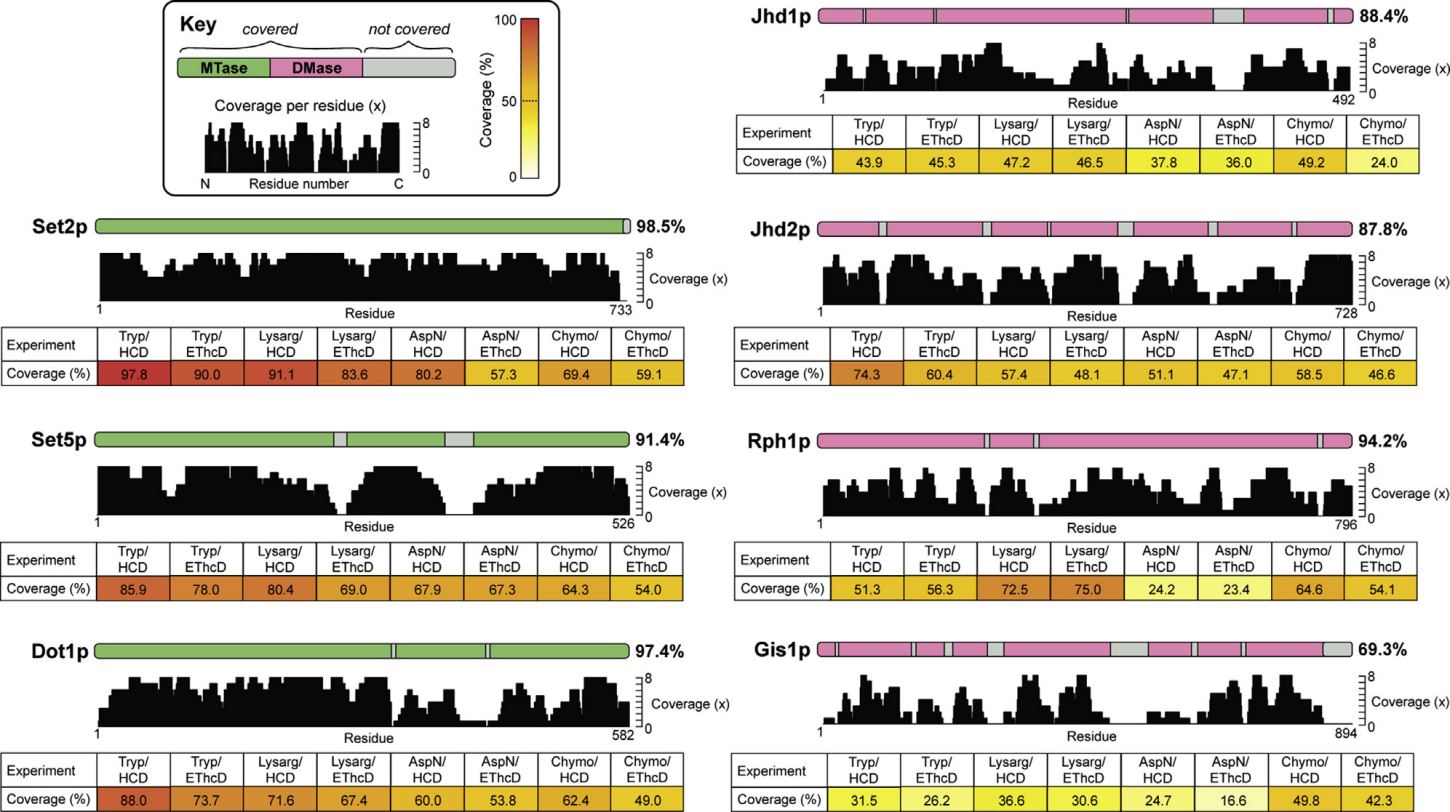
**Sequence coverage maps of histone MTase and DMase enzymes.** Homologously overexpressed and purified yeast histone MTases and DMases were subject to four separate proteolytic digestions (trypsin, LysargiNase, Asp-N, and chymotrypsin) and two mass spectrometric analyses with different fragmentation types (HCD and EThcD), giving eight experiments per enzyme. Residues covered in at least one mass spectrometry experiment are shown as colored bars along the linear protein maps (MTases in *green*, DMases in *pink*), while residues not covered are shown in *gray*. A histogram depicts the number of times each amino acid residue was covered across the eight experiments. The protein sequence coverage obtained for each protease/fragmentation method combination is tabulated per protein and colored according to the color scale (*top left*).

**Figure 2:**
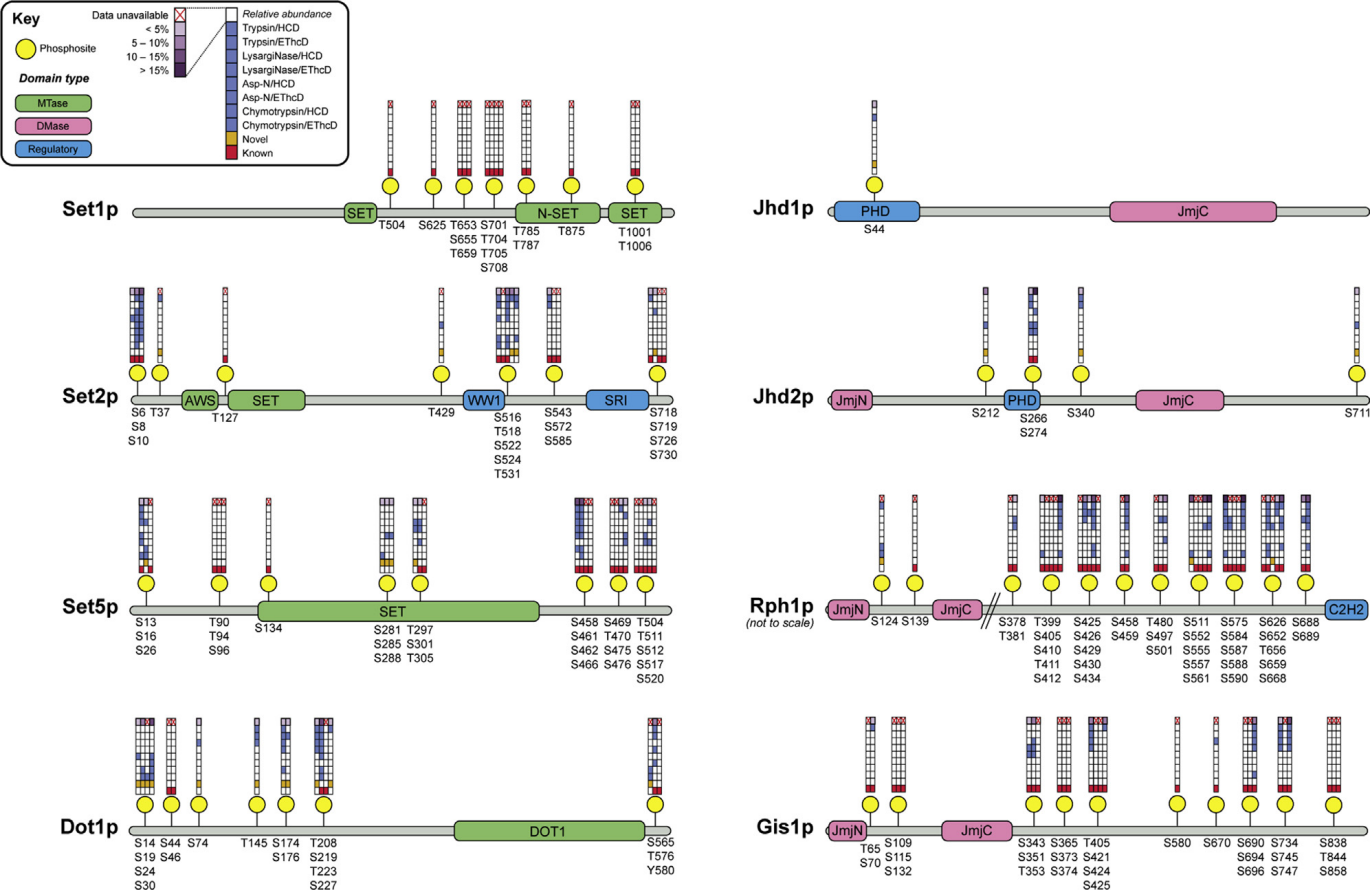
**Phosphorylation sites on yeast histone MTases and DMases.** Collation of phosphorylation sites identified in this study and from previous high-throughput phosphoproteomic analyses (Table S2). Phosphosites (*yellow*) are displayed to scale along linear protein sequence maps comprising MTase (*green*), DMase (*pink*), and other regulatory domains (*blue*). Grids above each phosphorylation site give details about the site’s identification and are arranged according to the key (*top left*). Phosphorylation sites that have previously been reported in the literature are denoted by a *red square*, while novel sites identified in this study are shown with a *gold square*. *Blue squares* illustrate the identification of phosphosites using different protease/fragmentation method combinations. The uppermost grid square denotes the relative occupancy of phosphorylation at a given residue, shaded in *purple* according to the color intensity scale (*top left*). Phosphosite abundance measurements were determined by mass spectrometric label-free quantification of phosphopeptides and their unmodified counterparts (Table S3). Where sites occur proximally to one another, grids are merged, and each column (*left to right*) corresponds to the phosphorylated residues listed below the protein map (*top to bottom*).

Our use of orthogonal techniques yielded high protein sequence coverage and extensive modification site identification (Fig. 1). In combination, the eight mass spectrometry experiments produced near-complete sequence coverage for four enzymes (>90%), high coverage for a further two enzymes (>85%), and 69% coverage for Gis1p, which was expressed and purified at lower levels (Fig. S1). Importantly, most regions of all proteins were detected *via* different methods, thus providing confirmatory evidence for the existence or absence of modification sites. For example, a total of 47 phosphorylation sites were identified in multiple independent mass spectrometry analyses (Fig. 2). Our data also showed that different protease/fragmentation method combinations were more effective for some enzymes than others; while trypsin achieved the highest coverage for most proteins, LysargiNase and chymotrypsin were optimal for Rph1p and Gis1p, respectively.

### Phosphorylation of histone MTases and DMases is widespread, but enzymes are phosphorylated to different extents

To establish the extent to which histone MTase and DMase enzymes are connected to the intracellular signaling network, mass spectrometry data were searched for the presence of phosphorylation. Putative phosphosites were filtered using stringent score and localization probability cutoffs, thus generating a high-confidence library of modification sites. From all analyses, we identified 180 non-redundant phosphopeptides (Table S1) mapping to 75 unique phosphorylation sites across seven histone MTase and DMase enzymes (Fig. 2). We confirmed 47 phosphosites from large-scale phosphoproteomic studies and report 28 novel sites. This expands the number of known phosphorylation sites on yeast histone methylation enzymes to 143 (Table S2). All phosphorylation sites, identified in this study or otherwise, are presented in Figure 2. Importantly, novel phosphorylation sites were often supported by orthogonal lines of experimental evidence. For example, the novel phosphorylation of Dot1p at threonine 208 was unambiguously identified by HCD fragmentation of both tryptic (Fig. S2*A*) and LysargiNase-generated (Fig. S2*B*) peptides. Similarly, novel phosphorylation at serine 30 of Dot1p was confirmed by exemplar HCD and EThcD fragmentation spectra of chymotryptic (Fig. S2*C*) and Asp-N-generated peptides (Fig. S2*D*), respectively.

With respect to the number of phosphorylation sites present on each enzyme, some striking trends emerged. Histone MTases Set1p, Set2p, Set5p, and Dot1p are all phosphorylated to similar degrees, harboring between 14 and 26 sites each (Fig. 2). By contrast, histone DMases are phosphorylated to markedly different levels. The paralogous H3K36 DMase enzymes Rph1p and Gis1p are both extensively phosphorylated, with 36 and 26 phosphosites, respectively, while Jhd1p and Jhd2p are phosphorylated at only one and five sites, respectively. Note that the low numbers of phosphosites on Jhd1p and Jhd2p are not just due to low sequence coverage (Fig. 1) or small protein size. Our results suggest that specific histone methylation enzymes are connected to the intracellular signaling network in very different ways. Enzymes with a large number of phosphosites (*e.g.*, Set5p, Rph1p, and Gis1p) are potential integrators of signaling information, while enzymes with few sites (*e.g.*, Jhd1p and Jhd2p) are likely to be regulated through other means.

We then explored the relative stoichiometry of phosphorylation for the enzymes, as purified from the yeast cell. This was done by label-free quantification of phosphopeptides and their unmodified counterparts (Table S3). Our quantitative analysis revealed that phosphorylation sites on histone methylation enzymes exist at low stoichiometry, ranging from <0.1% to 26% in abundance relative to corresponding unphosphorylated peptides (Fig. 2 and Table S3). These observations are generally consistent with absolute phosphorylation stoichiometry measurements across the human phosphoproteome, with the majority of sites existing at less than 20% occupancy (40), and studies that show that the majority of phosphosites in yeast are of ≤30% stoichiometry (41). The relatively low stoichiometry of phosphorylation in these enzymes suggests that the sites are not constitutive.

In regard to the relative amount of phosphorylation that is present at specific sites, we identified some modest but appreciable differences between histone MTase and DMase enzymes. Rph1p and Gis1p, histone DMases with the largest number of phosphosites (Fig. 2), also have the highest average relative occupancy of phosphorylation across their cognate sites, at 9.1% and 5.9%, respectively (Table S3). By contrast, histone MTases exhibit overall lower levels of phosphorylation, with Set5p phosphosites averaging only 2.4% in their relative abundance. These findings provide further evidence for the differential regulation of histone MTase and DMase enzymes by upstream signaling pathways.

### Histone MTases are predominantly phosphorylated within disordered sequences, while DMases are phosphorylated within ordered regions

**Figure 3:**
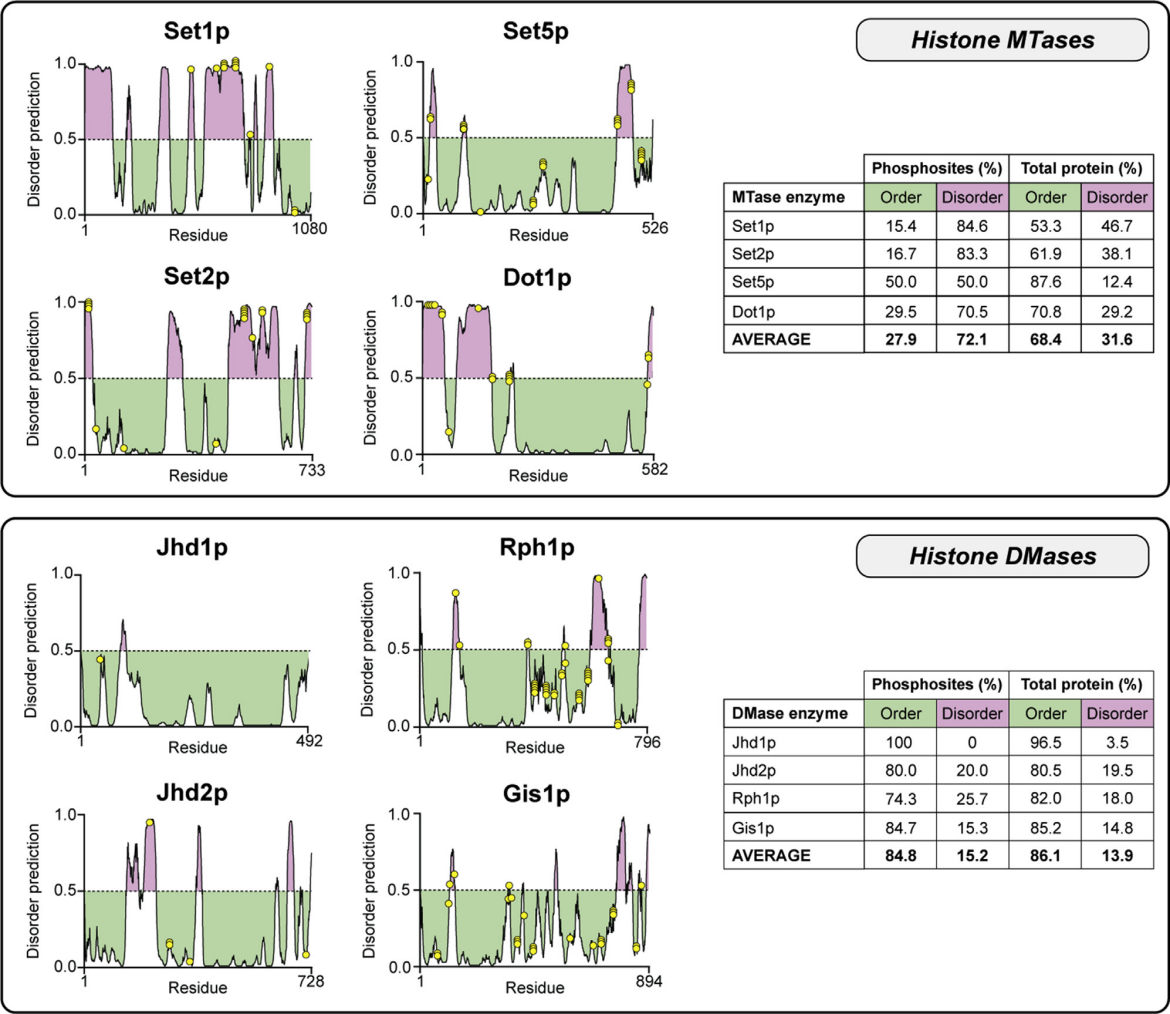
**Histone MTase and DMase enzymes are differentially phosphorylated within predicted regions of order and disorder.** DISOPRED3 was used to predict the propensity of local amino acid sequences, within histone MTase (*top*) and DMase (*bottom*) enzymes, to adopt an ordered or disordered conformation. Predicted regions of disorder are above the dotted midline and shaded in *purple*, while predicted ordered sequences are below the midline and shaded in *green*. Phosphorylated residues are shown as *yellow dots* along each disorder plot, and their occurrence within ordered and disordered regions is tabulated per protein. Histone MTases are predominantly phosphorylated in disordered regions while DMases tend to be phosphorylated in ordered stretches.

Having identified and collated a large number of phosphorylation sites on yeast histone MTase and DMase enzymes, we next examined their potential regulatory role by contextualization within relevant sequence and structural features. Firstly, phosphosites were mapped onto known protein domains (Fig. 2) and disorder prediction plots (Fig. 3). We found that there were very few phosphorylation sites located within catalytic, regulatory, or interaction domains of histone MTase and DMase enzymes. Of the 143 phosphorylation sites identified to date, only 15 (10.5%) resided in known domains (Fig. 2). In terms of catalytic domains, three phosphosites on Set1p were within its N-terminal pre-SET domain, and an additional two sites were found in its SET MTase domain. Set5p was the most heavily domain-phosphorylated enzyme, harboring seven phosphosites in its catalytic SET domain. No phosphorylation sites were identified on Jumonji N (JmjN) or Jumonji C (JmjC) DMase domains. With respect to regulatory and interaction domains, one phosphosite on Jhd1p (serine 44) and two sites on Jhd2p (serine 266 and serine 270) were found within chromatin interacting plant homeodomain (PHD) finger domains, while two phosphosites on Dot1p (serine 174 and serine 176) lie immediately adjacent to its DNA-binding motif. These phosphosites may dictate the capacity for Dot1p, Jhd1p, and Jhd2p to bind chromatin. Interestingly, phosphorylation sites at serine residues 458 and 459 of Rph1p are located within its bipartite nuclear localization signal and may therefore regulate nuclear import in a manner similar to nucleolar protein Npl3p, which is known to interact with karyopherin Mtr10p upon Sky1p-mediated phosphorylation (42).

Our analysis, importantly, revealed that histone MTases and DMases have demonstrably different disorder profiles; in terms of both total protein disorder and phosphosite distribution. This may reflect distinct modes of action and/or regulation. With the exception of Set5p, histone MTases are predicted to be more disordered than their DMase counterparts, ranging between 29% (Dot1p) and 47% (Set1p) sequence disorder (Fig. 3, *top panel*). Although histone MTases are still mostly ordered, their phosphorylation sites reside predominantly within disordered stretches. This enrichment of phosphorylation within disordered sequences on histone MTase enzymes is of functional relevance as such regions are known to modulate protein–protein interactions (43). For example, Set1p is the most disordered protein, in terms of both amino acid sequence and phosphosite occurrence, and is the only yeast histone methylation enzyme that forms a catalytically active complex *in vivo* (COMPASS; Set1C) (44). Of its 14 phosphosites, 85% are located in disordered regions and are particularly concentrated within a highly disordered sequence between residues 570 and 672. Multiple phosphorylation sites within intrinsically disordered regions are known to cooperatively cause order/disorder conformational transitions (45, 46) and the interactions of Set1p with Set1C subunits may involve a similar mechanism. In the case of Set2p, phosphorylation at serine 718 is located within a disordered sequence that physically contacts the phosphorylated C-terminal domain of RNA polymerase II and may therefore regulate interaction (47). By contrast with MTases, histone DMases are largely ordered proteins, with all four enzymes predicted to have at least 80% of their sequence in an ordered conformation (Fig. 3, *bottom panel*). Consistent with total protein values, the majority of phosphorylation sites (>70%) on DMase enzymes are located in ordered sequence contexts.

### Phosphorylation sites on Set1p and Dot1p are spatially adjacent to important structural features in three dimensions

**Figure 4:**
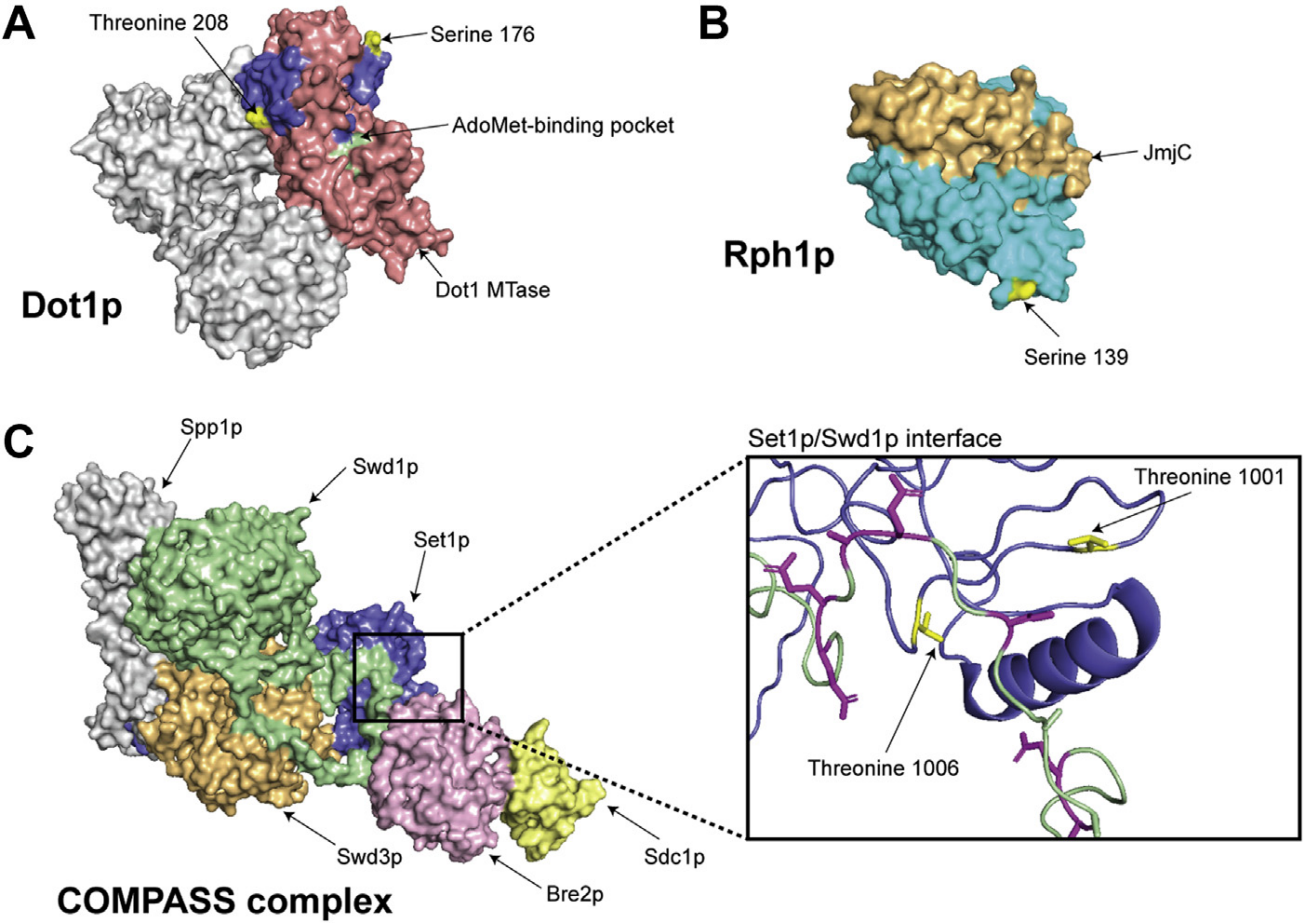
**Phosphorylation sites on yeast histone MTase and DMase enzymes, in the context of crystal structures.** Structures for four yeast histone MTase and DMase enzymes have been resolved to date. All structures were visualized with PyMOL. *A*, homotrimeric Dot1p structure (PDB ID: 1U2Z). An individual Dot1p monomer (*blue*) and its catalytic MTase domain (*pink*) are shown, while both other subunits are grayed out. Two novel phosphorylation sites identified at serine 176 and threonine 208 (*yellow*) lie structurally adjacent to the cofactor-binding pocket (*green*). *B*, partial Rph1p structure spanning its N-terminal 370 amino acid residues (PDB ID: 3OPW). A known phosphorylation site at serine 139 (*yellow*) is spatially exposed on the Rph1p structure (*cyan*) but is not located in its JmjC DMase domain (*gold*). *C*, multimeric structure of the yeast COMPASS complex (Set1C, PDB ID: 6BX3). The C-terminal 280 amino acid residues of MTase Set1p (*blue*) have been resolved. Other constituents of the complex include Bre2p (*pink*), Sdc1p (*light yellow*), Spp1p (*gray*), Swd1p (*green*), and Swd3p (*orange*), as well as accessory proteins Shg1p and Swe2p (not shown). *Inset*, phosphorylation sites on threonine residues 1001 and 1006 (*yellow*) of Set1p (*blue*) reside at its interaction interface with Swd1p (*green*). Negatively charged aspartate and glutamate residues on Swd1p (*magenta*) may be affected by proximal Set1p phosphorylation.

To determine whether phosphorylation is associated with interaction interfaces, phosphosites were projected onto three-dimensional crystal structures of histone MTases and DMases. Structures for four enzymes from *S. cerevisiae* (Set1p, Set2p, Dot1p, and Rph1p), have, at least partially, been resolved (Fig. 4). The structure for Set2p has not been included here as only its Set2 Rbp1 interacting (SRI) domain, which does not encompass any observed phosphosites, has been structurally determined. Our contextualization revealed that phosphorylation at threonine 208 of Dot1p is located close to the interaction interface between adjacent Dot1p monomers, which comprise the catalytically active trimeric molecule (Fig. 4*A*). Given that phosphorylation is known to regulate multimerization of some yeast protein MTases (*e.g.*, Hmt1p (48)), phosphorylation at this site may control Dot1p trimer assembly. The partial structure of Rph1p is limited to its N-terminal 370 residues, whereas the majority of its phosphosites are clustered toward its C-terminus, meaning that only a single phosphorylation site at serine 139 could be structurally mapped (Fig. 4*B*). This phosphosite is surface exposed and located on a spatially accessible protrusion distal to the catalytic JmjC domain.

Set1p is the catalytic constituent of the multi-subunit COMPASS, or Set1C, H3K4 MTase complex, for which the molecular stoichiometry and three-dimensional configuration are known (Fig. 4*C*). Notably, two phosphorylation sites previously reported on Set1p (35), at threonine 1001 and threonine 1006, are situated at its interface with Swd1p (Fig. 4*C*, *inset*). The region on Swd1p that physically contacts Set1p is rich in negatively charged aspartate and glutamate residues, thus Set1p phosphosites could impede Swd1p binding and subsequent Set1C assembly.

### Some phosphorylation sites on yeast histone MTase and DMase enzymes are conserved in mammalian orthologs

**Figure 5:**
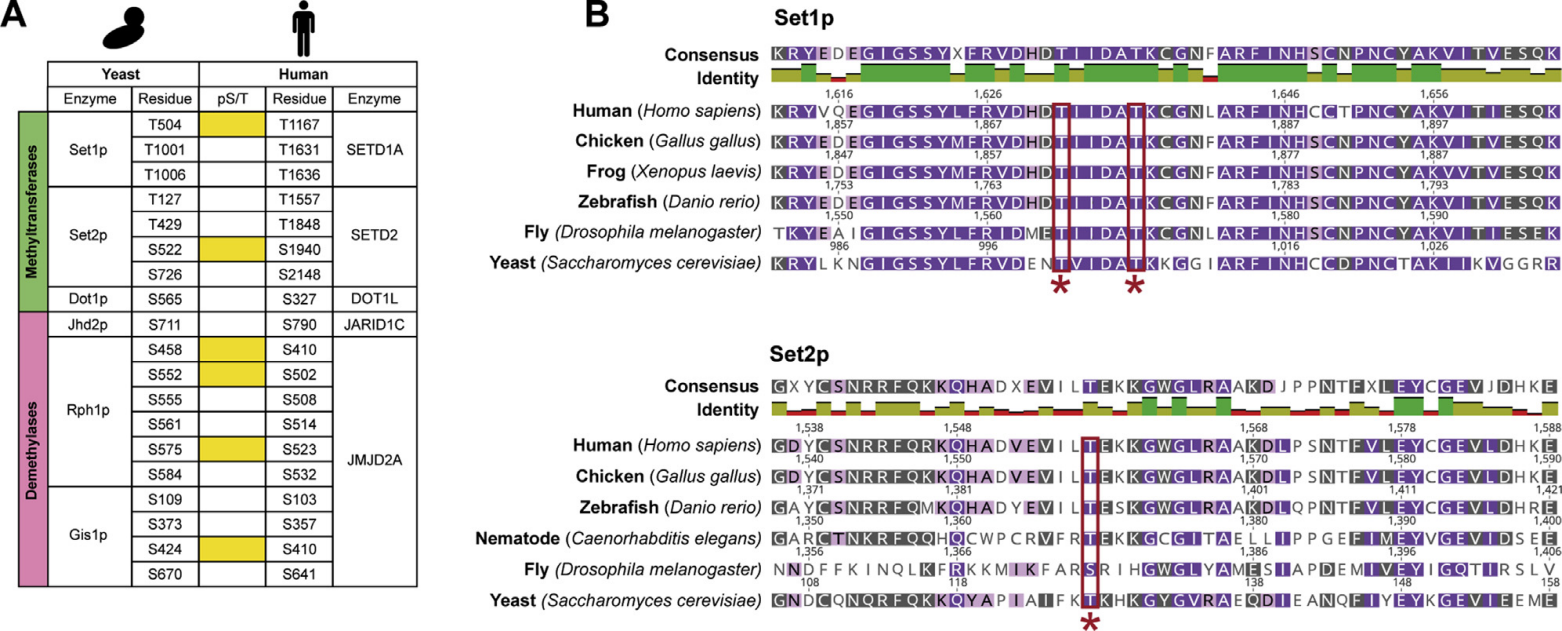
**Conservation of yeast phosphorylation sites on orthologous histone MTase and DMase enzymes.***A*, pairwise protein sequence alignments were performed between all yeast histone MTase and DMase enzymes and their respective human orthologs, with the exception of Set5p, which is not conserved in mammalian cells. Residues that are phosphorylated in *S. cerevisiae* and are conserved at the sequence level in human are tabulated. Conservation of phosphorylation at these residues is shown by *yellow squares*. *B*, representative portions of multiple sequence alignments for yeast Set1p (*top*) and Set2p (*bottom*) with several eukaryotic orthologs. Conservation of chemically similar amino acid residues is as follows: 100% = *violet*, 80 to 100% = *mauve*, 60 to 80% = *khaki*, < 60% = *white*. Residue numbers for each species are shown above their respective sequences. Instances where a phosphorylated serine or threonine residue on a yeast protein is conserved across all organisms are denoted by a *red box* and *asterisk* below the alignment. Sequence alignments were generated in Geneious Prime (version 2020.1.2).

We explored the evolutionary conservation of phosphoresidues on histone MTase and DMase enzymes in eukaryotic orthologs. To determine the degree of conservation in mammals, pairwise sequence alignments were performed between all yeast histone MTases and DMases and their equivalent human proteins (Fig. 5*A*), with the exception of the yeast-specific MTase, Set5p. Strikingly, of the 19 phosphorylated amino acids in yeast that are conserved at the sequence level in human, six are known to be phosphorylated *in vivo* (Fig. 5*A*) (49, 50). For example, phosphorylation at serine 522 of yeast Set2p is conserved at its corresponding residue in human, serine 1940 of SETD2. The retention of yeast phosphorylation sites in mammalian systems is of functional relevance as conserved sites are known to serve important roles in cellular signaling (51). More broadly, we found that amino acid residues, subject to phosphorylation in yeast, are located within exquisitely conserved regions of orthologous proteins in human and in other eukaryotes, for which robust phosphoproteomic data is currently unavailable. For example, threonine residues 1001 and 1006 of Set1p and threonine 127 of Set2p, all known to be phosphorylated in yeast, are invariant within highly homologous sequences in several eukaryotic orthologs (Fig. 5*B*).

### A draft regulatory network of histone methylation in *S. cerevisiae*

**Figure 6:**
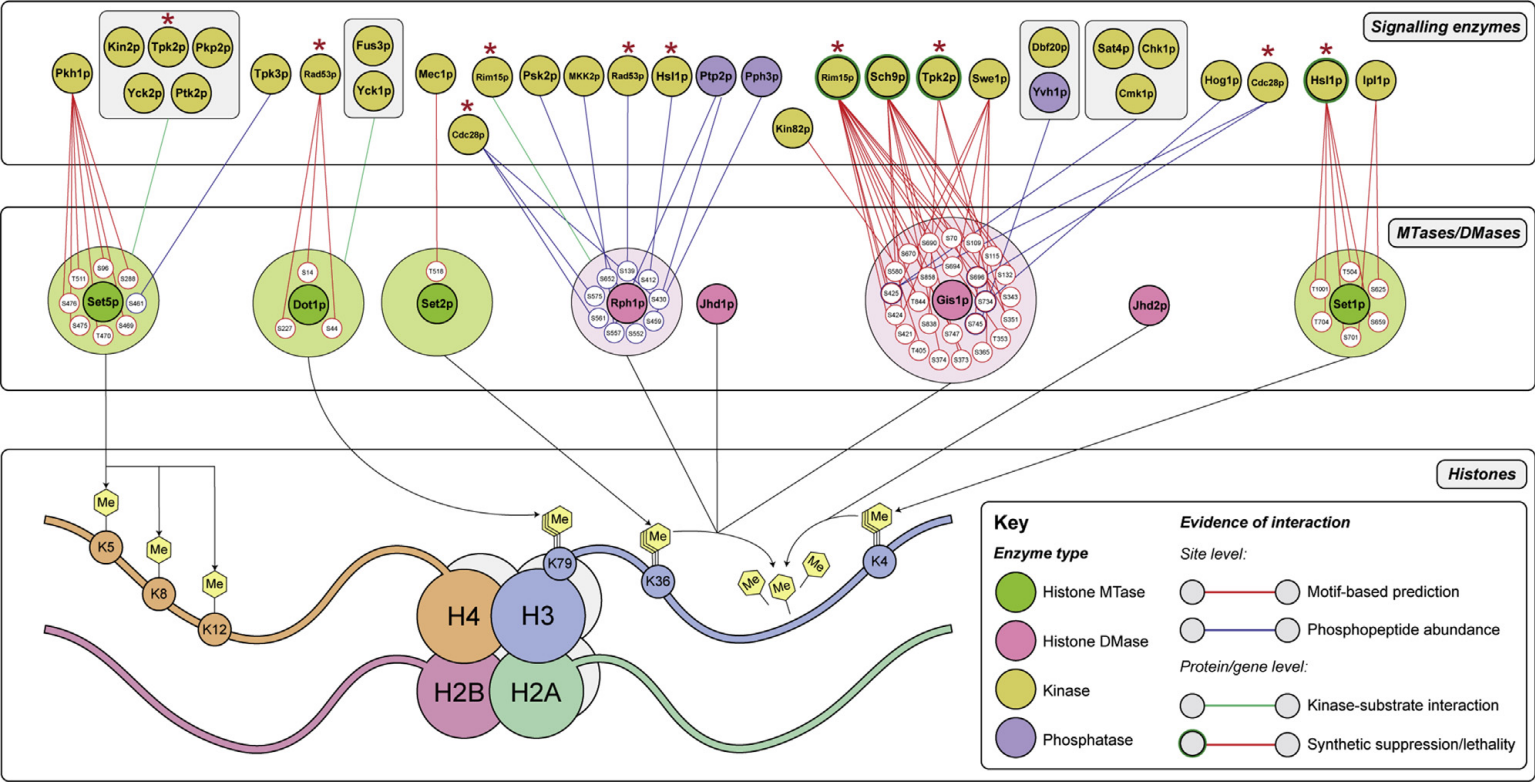
**Draft regulatory network of histone methylation in *S. cerevisiae* showing phosphosites with a known or predicted upstream kinase or phosphatase.** Model of the possible connections between the yeast histone methylation system and the intracellular signaling network. Histone MTase (*green*) and DMase (*pink*) enzymes and their respective histone targets are shown in the *middle/lower panels*. Known phosphorylated residues with putative links to upstream kinases (*yellow*) or phosphatases (*purple*) are displayed around their cognate histone MTase/DMase enzyme. For ease of visualization, only phosphosites with a known or predicted relationship with an upstream signaling protein are shown. Potential interactions between phosphosites and upstream regulators are shown as edges in the *upper/middle panels* and colored by evidence type according to the key (*bottom right*). Motif-based kinase prediction (*red*) was performed using NetworKIN3.0 (52), while quantitative phosphopeptide data (*blue*) were collected from Bodenmiller *et al.* (53) and Holt *et al.* (54). At the protein level, putative kinase–substrate interactions (*green*) were from Ptacek *et al.* (55). Where at least one other line of evidence was available for a connection, genetic relationships (*e.g.*, synthetic suppression, lethality) were obtained from the Yeast Kinase Interaction Database (56) and depicted by a *green border* around kinase nodes. Instances where a single kinase is predicted to interact with multiple histone methylation enzymes are shown by a *red asterisk*. While provisional, our network reveals a highly interconnected and integrated regulatory structure for histone methylation in yeast and highlights the diverse signaling pathways that may transmit information to histone MTases and DMases to regulate enzyme function.

There is a paucity of kinases known to phosphorylate specific sites on histone MTase and DMase enzymes in yeast. As such, our understanding of the biological function of phosphosites and the network context of their cognate proteins is fragmentary. Using publicly available phosphoproteomic datasets, interaction databases, and prediction tools, we modeled possible connections between the histone methylation enzymes and cellular signaling pathways (Fig. 6). Our draft regulatory network incorporates motif-based kinase predictions (52), quantitative phosphopeptide data (53, 54), as well as known physical (55) and genetic (56) kinase–substrate interactions. It establishes provisional regulatory relationships between specific enzymes, phosphosites, and their upstream signaling proteins. The regulatory model proposed here will help inform future targeted studies into histone methylation and its phosphoregulation.

The degree to which histone MTases and DMases are likely connected to the intracellular signaling network varies considerably between enzymes. Consistent with the different amounts of phosphorylation present on histone DMases, Rph1p and Gis1p are highly connected to signaling pathways while Jhd1p and Jhd2p do not have any known or predicted interactions with kinases or phosphatases (Fig. 6). Gis1p is the most signaling-connected enzyme; 25 of its 26 phosphosites are predicted to be recognized by at least one kinase, and many of such predictions were supported by complementary genetic evidence (*e.g.*, Rim15p, Sch9p, and Tpk2p). Interestingly, our network analysis revealed a highly interconnected and integrated regulatory structure whereby kinases may phosphorylate multiple sites on the same or different enzymes. For example, seven observed phosphosites on Set5p are predicted to be regulated by the sphingolipid signaling kinase, Pkh1p, and five sites on Set1p are likely phosphorylated by Hsl1p, a mitogen-activated protein kinase (Fig. 6). There are also several instances where a single kinase may act on multiple enzymes. Dot1p and Rph1p are both predicted to be phosphorylated by Rad53p, a DNA damage response kinase, while Set5p and Gis1p have possible regulatory links to the cAMP-dependent signaling kinase, Tpk2p (Fig. 6). The range of different kinases presented in our regulatory network are constituents of diverse signal transduction pathways, thus highlighting the potential complexity of the phosphoregulation and signal responsiveness of histone methylation in yeast.

### A phosphorylation cluster within an acidic and intrinsically disordered N-terminal region of MTase Set2p regulates H3K36 methylation levels *in vivo*

**Figure 7:**
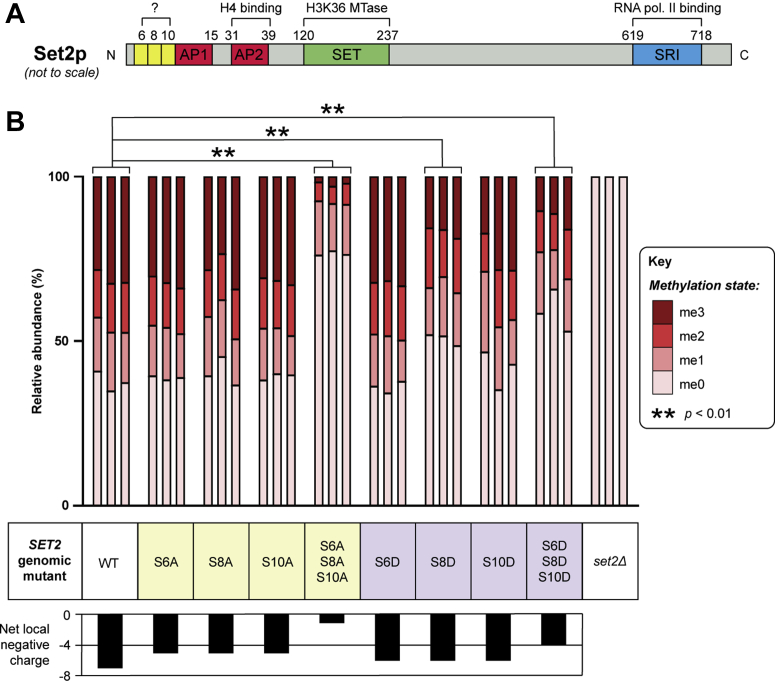
**Phosphorylation within an N-terminal intrinsically disordered region of MTase Set2p regulates H3K36 methylation levels *in vivo*.***A*, schematic of Set2p sequence features. Phosphorylation sites (*yellow*) lie immediately adjacent to an acidic patch at residues 11 to 15 (AP1; sequence EDEKE). A similar downstream acidic patch at residues 31 to 39 (AP2; sequence DQEPDLTEE) is known to regulate Set2p histone H4-binding affinity and is required for its H3K36 MTase activity (58). For simplicity, the AWS domain shown in Figure 2 has been omitted. *B*, mutagenesis of Set2p phosphorylation sites affects H3K36 methylation *in vivo*. Phosphonull (serine-to-alanine; *yellow*) and phosphomimetic (serine-to-aspartate; *lilac*) mutations of Set2p phosphorylation sites, either alone or in combination, were engineered into the *S. cerevisiae* chromosome. For each mutant, the net local negative charge within the N-terminal 15 amino acid residues of Set2p, after considering phosphorylation and charge changes due to mutation, is depicted as a bar chart. H3K36 methylation levels in wild-type (WT) and *SET2* genomic mutant yeast strains were quantified by label-free mass spectrometric analysis of a triply-charged, K36-containing tryptic peptide (sequence KSAPSTGGV**K**KPHR), in its unmodified (*m/z* = 539.97; me0, triply propionylated), monomethylated (*m/z* = 544.65; me1, triply propionylated), dimethylated (*m/z* = 530.64; me2, doubly propionylated), and trimethylated (*m/z* = 535.31; me3, doubly propionylated) forms (see Fig. S3 for extracted ion chromatograms). The *in vivo* distribution of H3K36 methylation states across the different mutants is visualized as a stacked bar chart with *n* = 3 biological replicates. A *SET2* deletion strain (*set2Δ*) was included as a control, confirming ablation of all H3K36 methylation states upon methyltransferase knockout. Statistical comparisons between each mutant and the wild-type control (∗∗*p* < 0.01 *versus* WT) were carried out using an ordinal logistic regression model with a proportional odds assumption (see Fig. S4 for details).

We next investigated whether phosphorylation sites identified or confirmed here could affect the function of histone methylation enzymes *in vivo.* Three phosphorylation sites on H3K36 MTase Set2p, at serine residues 6, 8, and 10 (Fig. 2), were of special interest as they are in a region of intrinsic disorder (Fig. 3) and thus constitute a putative regulatory cluster. The sites are adjacent to an EDEKE acidic patch, at residues 11 to 15 (Fig. 7*A*), that may function cooperatively with phosphorylation to regulate Set2p activity through an electrostatic mechanism. Accordingly, multiple phosphorylation sites at the N-terminus of mammalian heterochromatin protein 1α are known to regulate its recruitment to chromatin (57). In Set2p, a downstream acidic patch of sequence DQEPDLTEE, between residues 31 and 39, has been reported as a histone H4 interaction motif that is required for nucleosomal binding and H3K36 MTase activity (58).

For functional characterization of Set2p, phosphonull (serine-to-alanine) and phosphomimetic (serine-to-aspartate) mutations of phosphosites were engineered into the *SET2* genomic locus by homologous recombination (59). Six strains with single amino acid mutations of Set2p were generated, namely S6A, S8A, S10A, S6D, S8D, and S10D, and triple phosphonull and triple phosphomimetic strains of S6A/S8A/S10A and S6D/S8D/S10D, respectively, were also generated. We then quantified the effects of these mutations on *in vivo* levels of H3K36 methylation by mass spectrometry (Fig. S3). In yeast cells expressing wild-type Set2p, H3K36 is mono-, di-, and tri-methylated at ∼15%, ∼15%, and ∼30%, respectively, and ∼40% is unmethylated (Fig. 7*B*). By comparison, a *set2Δ* negative control showed complete loss of all H3K36 methylation states (Fig. 7*B* and Fig. S3). Phosphonull and phosphomimetic mutations of Set2p phosphosites had different effects on H3K36 methylation levels. Using an ordinal logistic regression model with a proportional odds assumption (Fig. S4*A*), the overall H3K36 methylation of each *SET2* mutant yeast strain was compared with the wild-type control. This revealed significant differences for triple phosphonull (*p* = 2.0 × 10^−16^), triple phosphomimetic (*p* = 2.2 × 10^−9^), and S8D (*p* = 1.1 × 10^−4^) Set2p phosphomutants when compared with the wild-type control (Fig. 7*B*). These changes were characterized by a lower level of H3K36 trimethylation and a higher level of unmodified H3K36. Other mutants, however, did not show any statistically significant differences in H3K36 methylation.

Given that the degree of negative charge in the N-terminal region of Set2p may regulate its interaction with histones, we examined the net possible charge on amino acids 1 to 15 of Set2p, with and without mutations, and its association with H3K36 methylation. We considered one positive charge each for the N-terminus and lysine 3, six potential negative charges from phosphorylation sites at serine residues 6, 8, and 10, and one positive and four negative charges within acidic patch EDEKE. From this, wild-type Set2p can carry a net charge of −7 in its N-terminal region, while Set2p phosphomutants have a reduced capacity to hold negative charge (60). With respect to phosphonull substitutions, single-site mutations (S6A, S8A, S10A; net charge of −5) did not appreciably modulate H3K36 methylation levels, while simultaneous mutation of all three sites (S6A/S8A/S10A; net charge of −1) significantly decreased overall methylation stoichiometry, with only ∼2% of H3K36 being trimethylated (Fig. 7*B*). This observation suggests that there is a minimum charge requirement within this region for Set2p to retain wild-type levels of activity. With respect to phosphomimetic substitutions, each confers a single, but not double negative charge. Accordingly, single-site mutations (S6D, S8D, S10D; net charge of −6) did not markedly affect H3K36 methylation, while the triple phosphomimetic substitution (S6D/S8D/S10D; net charge of −4) reduced the relative abundance of H3K36 trimethylation to ∼12% (Fig. 7*B*). This model highlights the likely importance of charge in the N-terminus of Set2p, as the measured methylation of H3K36 in triple phosphomimetic and triple phosphonull mutations corresponded to the capacity for these mutant proteins to carry negative charge.

### Histone MTase and DMase enzymes are also post-translationally acetylated and ubiquitinated

**Figure 8:**
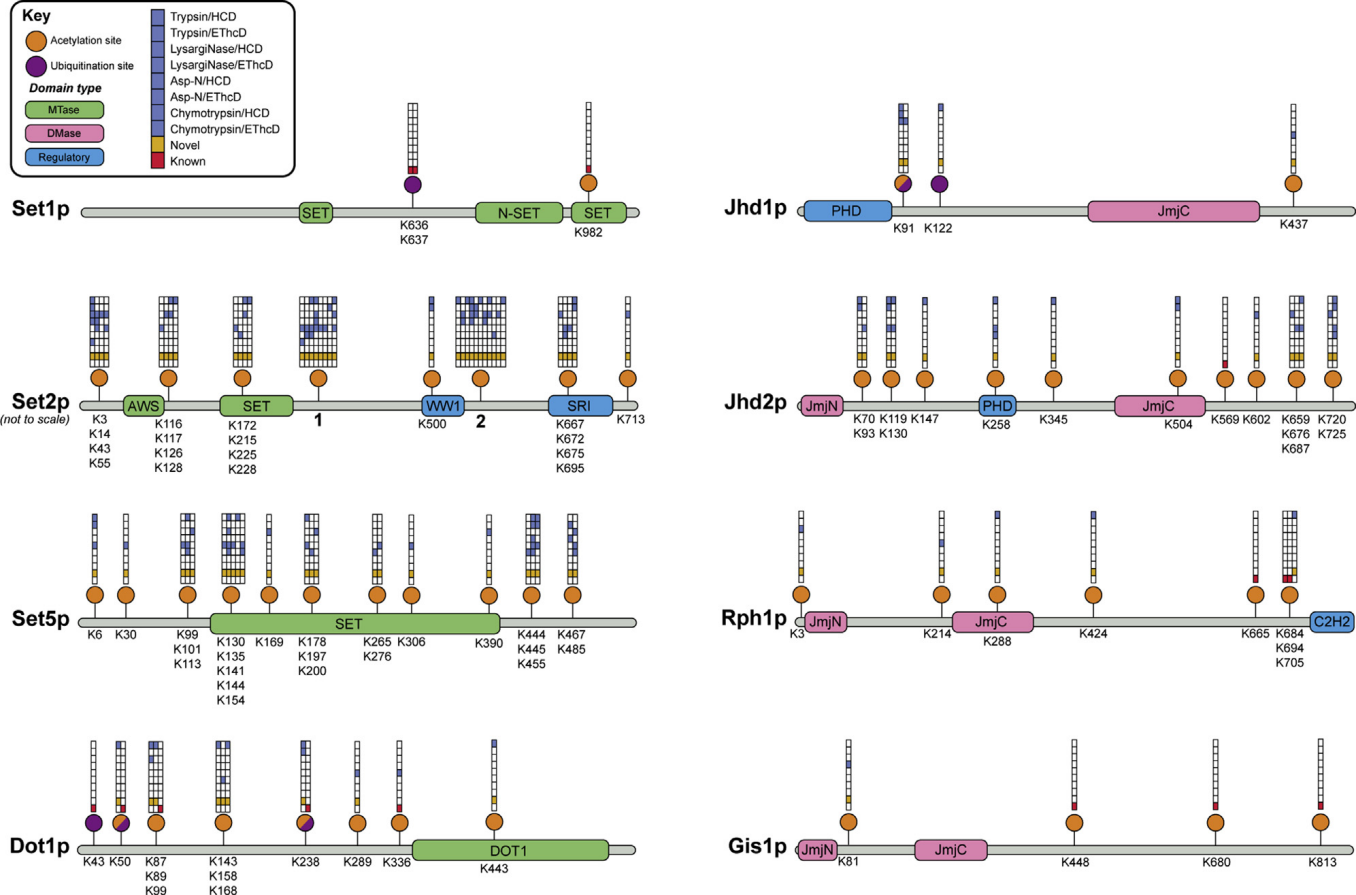
**Acetylation and ubiquitination sites on yeast histone MTases and DMases.** Collation of acetylation and ubiquitination sites identified in this study and from previous high-throughput proteomic analyses (Tables S4 and S5). Acetylation (*orange*) and ubiquitination (*purple*) sites are displayed along linear protein sequence maps comprising MTase (*green*), DMase (*pink*), and other regulatory domains (*blue*). Sites that have evidence for both acetylation and ubiquitination are depicted as *half orange*/*half purple circles*. Grids above each PTM site give details about the site’s identification and are arranged according to the key (*top left*). Sites that have been previously reported in the literature are denoted by a *red square*, while novel sites identified in this study are shown with a *gold square*. *Blue squares* illustrate the identification of PTM sites using different protease/fragmentation method combinations. Where sites occur proximally to one another, grids are merged, and each column (*left to right*) corresponds to the modified residues listed below the protein map (*top to bottom*). To accommodate the large number of modification sites, Set2p is not illustrated to scale, and residue labels have been removed in instances where more than five grids have been merged (see numbering). These numbered grids correspond to the following residues from *left to right*: 1 = K329, K340, K412, K428, K433, K447, K450, K459; 2 = K510, K530, K541, K566, K574, K578, K584, K598, K602, K607, K620.

Having identified extensive post-translational phosphorylation of yeast histone MTases and DMases, we sought to establish the extent to which enzymes are subject to other modification types. Akin to our systematic approach to phosphosite discovery, combinatorial mass spectrometry data were queried for the presence of acetylation, methylation, ubiquitination, SUMOylation, ADP-ribosylation, and crotonylation. Altogether, we identified 92 acetylation sites across all enzymes that were analyzed and discovered two ubiquitination sites on Jhd1p (Fig. 8). Our analysis did not provide evidence for the presence of any other modification types that were examined. All acetylated and ubiquitinated residues on yeast histone MTase and DMase enzymes, identified in this study or otherwise, are summarized in Figure 8. Our modification sites add to the ten acetylation sites (Table S4) and four ubiquitination sites (Table S5) that have previously been identified across five enzymes, as well as SUMOylation of Set1p, which could not be localized to a specific residue (38).

With respect to acetylation, many sites identified here were verified by orthogonal mass spectrometric analyses. We note that only one acetyl site (lysine 336 of Dot1p) confirms previous reports; however, the number of proteome-scale studies of protein acetylation in *S. cerevisiae* is modest. Similar to the distribution of phosphorylation across enzymes, different histone MTases and DMases are also acetylated to markedly different extents. Three-quarters of all acetylation sites are found on just three enzymes: Set2p, Set5p, and Jhd2p, which harbor 37, 23, and 15 sites, respectively. Mapping of sites onto linear protein sequences revealed that acetylation generally occurs outside of MTase and DMase domains. Of the 101 acetylation sites identified to date, 27 (26.7%) are located within catalytic or regulatory domains (Fig. 8). It is interesting that extensively phosphorylated enzymes are not necessarily acetylated to the same degree, and vice versa. For example, histone DMases Rph1p and Gis1p harbor the largest number of phosphosites (Fig. 2) but show a relatively modest level of acetylation (Fig. 8). Conversely, Jhd2p is extensively acetylated but minimally phosphorylated.

Ubiquitination of histone MTase and DMase enzymes is substantially less prevalent than phosphorylation and acetylation, and ubiquitin sites are exclusively found outside of protein domains. Our discovery of two ubiquitination sites at lysine residues 91 and 122 of Jhd1p (Fig. S5) brings the total number of sites across the eight yeast histone methylation enzymes to seven (Fig. 8). Interestingly, lysine 91 is also acetylated *in vivo*, suggesting that there may be interplay between two competing modifications of the same residue. More broadly, it will be interesting to determine whether closely positioned modifications of different types participate in crosstalk on histone MTase and DMase enzymes. For example, three acetylation sites are proximal to phosphorylation at serine 572 of Set2p and may affect kinase recognition (Fig. S6). Moreover, two phosphorylation sites on Dot1p are located immediately adjacent to a known ubiquitination site at lysine 43 and may cooperatively comprise a phosphodegron (61).

## Discussion

Despite the central role of histone MTase and DMase enzymes in transcriptional regulation, no systematic characterization of their PTMs has been performed in eukaryotes to date. Here, we report the detailed analysis of PTM types and sites on all histone MTase and DMase enzymes in *S. cerevisiae* and show that they carry 75 phosphorylation sites, 92 acetylation sites, and two ubiquitination sites. Our comprehensive and quantitative phosphosite analysis revealed that phosphorylation sites on histone methylation enzymes exist at low stoichiometry in the yeast cell (40), suggesting that these sites are not likely to be constitutive. We found that specific enzymes are phosphorylated to markedly different degrees and therefore connected to the intracellular signaling network in different ways. Extensively phosphorylated enzymes, such as Set5p, Rph1p, and Gis1p, are likely to be integrators of signaling information, while enzymes with a small number of phosphosites, such as Jhd1p and Jhd2p, may be primarily regulated through other means, for example, at the level of transcription (reviewed in (24)). Phosphorylation was largely absent on catalytic and regulatory domains but enriched within predicted regions of disorder on histone MTases, highlighting a potential role in the modulation of protein–protein interactions. Indeed, we show that a phosphorylation cluster within an acidic and intrinsically disordered N-terminal region of Set2p affects its H3K36 MTase activity *in vivo.* Significantly, many phosphorylation sites on yeast histone MTases and DMases are conserved on human orthologs, and sequences containing phosphosites also exhibit exquisite amino acid conservation among eukaryotes. With respect to other modifications, our data show no evidence for the presence of protein methylation, ADP-ribosylation, SUMOylation, or crotonylation, suggesting that these PTMs do not have a role in the histone methylation system in yeast.

Histone methylation enzymes are phosphorylated to strikingly different extents. They are thus likely to be regulated through different mechanisms. This was particularly apparent for the two evolutionarily related pairs of histone DMases in yeast. The paralogous H3K36 DMases, Rph1p and Gis1p, are heavily phosphorylated, harboring 36 and 26 phosphosites, respectively, suggesting regulation at the post-translational level by upstream signaling pathways. Rph1p and Gis1p also have the highest average relative occupancy of phosphorylation across their cognate sites, thus furthering evidence for their extensive phosphoregulation. By contrast, the Jumonji domain-containing DMases, Jhd1p and Jhd2p, receive relatively little information from the intracellular signaling network, containing just one and five phosphosites, respectively. We note that the small number of sites was not just due to differences in protein overexpression or small protein size. The phosphosites on Jhd1p and Jhd2p may function as molecular switches to activate/inactivate enzyme function in response to a specific signal, or alternatively, transcriptional (62) and post-transcriptional (63) regulatory programs may largely govern Jhd1p and Jhd2p activity. Our observations in yeast are consistent with the mammalian histone methylation system, wherein specific MTase and DMase enzymes are phosphorylated to different degrees and are connected to and regulated by signal transduction pathways in different ways (24).

We have modeled a regulatory network of histone methylation, from the system-wide characterization of histone MTase and DMase enzymes. This model revealed an integrated structure whereby the methylation enzymes are connected to many different upstream signaling pathways. It is apparent that a single kinase (*e.g.*, Rad53p, Tpk2p, Cdc28p, and Rim15p) may have the capacity to transmit information to multiple histone methylation enzymes, raising the possibility of coordinated regulation. While evidence for kinases acting on multiple MTases and/or DMases in the histone methylation network has not been demonstrated in yeast, there are several instances of this in the human cell. For example, Akt kinase phosphorylates H3K36 MTase NSD2 (28) and H3K4 DMase JARID1A (64), while JAK2 targets both H3K27 MTase EZH2 (65) and H3K9 DMase JMJD1A (66). A pressing question is thus whether multiple enzymes and therefore histone methylation sites are coregulated in response to specific signals to orchestrate changes in gene expression. Our model also highlights the diversity of signaling kinases and pathways that are predicted to regulate the histone methylation network. These pathways, such as the cyclin-dependent, mitogen-activated, and DNA damage response signaling cascades, are not unexpected as they are known to be associated with changes in mammalian chromatin (25, 27, 67, 68, 69).

In the histone methylation enzymes, we found evolutionary conservation of phosphorylation sites and/or sequences subject to phosphorylation. This is a striking observation as highly conserved phosphosites are likely to be of critical function, particularly within the context of cellular signaling (51). A total of six phosphosites on yeast histone MTases and DMases were present in human orthologs (49, 50) and will be excellent targets for functional analysis. Specifically, phosphorylation at threonine 504 of yeast Set1p is conserved in its orthologous human and mouse proteins (SETD1A; threonine 1667 for *H. sapiens*, threonine 1774 for *M. musculus*) (70, 71). Moreover, three phosphosites on Rph1p and one site on Gis1p are conserved on their shared mammalian ortholog, JMJD2A (72, 73). The same phosphosites are likely to be conserved in other eukaryotes; however, this cannot be currently confirmed due to a lack of relevant phosphoproteomic data. We found a further 13 instances where a phosphorylatable amino acid residue, known to be modified in yeast, is conserved at the sequence level in mammals. The exquisite conservation of phosphosites and residues on orthologous enzymes suggests, as with many of the methylation sites on histones, that aspects of the histone methylation regulatory network may be evolutionarily conserved.

The molecular interactions of histone MTases may be regulated by phosphorylation. Sites on MTases were strongly enriched within disordered regions and at interaction interfaces but largely absent or under-represented on domains. This is functionally important as intrinsically disordered regions are known to mediate protein–protein interactions (74) and associations with nucleic acids (75, 76). The positioning of phosphorylation observed here is also consistent with our previous study of phosphorylation on five non-histone protein MTases in yeast (39). Evidence that phosphorylation may modulate interactions is that it is proximal to several important interaction interfaces on histone MTases, both in sequence and in three-dimensional space. Phosphorylation at serine 718 of Set2p lies within a region that physically contacts the phosphorylated C-terminal domain of RNA polymerase II (47), while phosphosites at serine residues 174 and 176 of Dot1p are immediately adjacent to its DNA-binding motif. Moreover, within the multimeric architecture of the H3K4 MTase complex, Set1C (77), two phosphorylation sites at threonine residues 1001 and 1006 of Set1p are spatially adjacent to aspartate and glutamate residues on Swd1p and may therefore regulate Swd1p binding and subsequent Set1C assembly through a charge-based mechanism (78).

Given the aforementioned differences in the extent to which histone methylation enzymes are phosphorylated, their cognate phosphosites are likely to have different functional roles. In the case of extensively phosphorylated proteins, such as Set5p, Rph1p, and Gis1p, an obvious question emerges: do individual phosphosites carry out distinct functional roles (switch), or does the cumulative effect of multiple phosphosites fine-tune enzyme activity (dial)? There is evidence emerging in support of both regulatory processes. Several targeted studies into histone DMases Rph1p and Gis1p have shown that specific phosphorylation events can have switch-like effects on function. For example, phosphorylation at serine 652 of Rph1p triggers its dissociation from chromatin in response to DNA damage (79), while phosphorylation at serine 425 of Gis1p controls its binding at promoters of nutrient-related genes (80). By contrast, the catalytic activity and chromatin association of histone H4 MTase Set5p are cooperatively regulated by ten phosphosites on its C-terminal region, rather than the presence or absence of specific sites (30), thus suggesting a dial-like regulatory mechanism. Looking ahead, it will be critical to establish whether the regulation of extensively phosphorylated histone MTases and DMases involves on/off switching in response to a specific signal, or whether it involves fine-tuning by multiple signals from independent signaling pathways. This will also establish whether some histone methylation enzymes are major integrators of signaling information (81).

Through mutagenesis studies, we have shown that a phosphorylation cluster at serine residues 6, 8, and 10 of Set2p, within an acidic and intrinsically disordered N-terminal region, affects its H3K36 MTase activity *in vivo.* Charge-based interactions are known to regulate the nucleosomal association of *S. cerevisiae* histone methylation enzymes (58, 82). An acidic patch between residues 31 and 39 (sequence DQEPDLTEE) is essential for Set2p to bind histone H4 and subsequently methylate H3K36 (58). Our results point to the existence of an additional regulatory region, comprising a phosphorylation cluster at serine residues 6, 8, and 10, and an acidic patch between residues 11 and 15 (sequence EDEKE). While this patch has previously been shown to be dispensable for Set2p nucleosomal binding and H3K36 MTase activity (58), that study did not consider phosphorylation. Adjacent phosphorylation at serine residues 6, 8, and/or 10 would increase the net negative charge within this N-terminal region and could control Set2p interactions with chromatin and thus function. Structural studies of *Chaetomium thermophilum* Set2 have shown that Set2 makes extensive contact with histone proteins H2A and H3 (83); however, the interaction of the disordered Set2 N-terminus with the nucleosome could not be resolved. We propose that the N-terminus of Set2p represents a “tuneable” interaction interface that receives multiple inputs from upstream signaling pathways *via* phosphorylation. This model contrasts with the downstream acidic patch in Set2p (residues 31–39) that is sequence encoded and thus not tuneable nor signal responsive.

In addition to phosphorylation, histone MTase and DMase enzymes in yeast are subject to post-translational acetylation and ubiquitination. With respect to acetylation, we found that proteins are modified to different degrees and that acetyl sites show clearer domain association than phosphorylation sites. This suggests that acetylation and phosphorylation may regulate enzyme function in distinct ways. Histone MTases Set2p and Set5p had a large number of acetyl sites and harbored acetylation clusters within their respective SET MTase domains. In addition, four acetylation sites on Set2p were located within its SRI domain, which directly interacts with the phosphorylated C-terminal domain of RNA polymerase II and is required for Set2p recruitment to actively transcribed chromatin (47). Importantly, acetylation of yeast and human histone methylation enzymes is known to regulate their catalytic activity (84) and protein interactions (85). However, given the complexities of protein acetylation, our data was not able to distinguish acetyltransferase-mediated sites from non-enzymatic acetylation events resulting from the spontaneous reaction of acetyl-CoA with exposed lysine residues (86). In terms of ubiquitination, we identified two novel sites on Jhd1p at lysine residues 91 and 122, adding to the five sites documented in the literature across all yeast histone methylation proteins. Intriguingly, lysine 91 was also found to be acetylated, suggesting that competition between ubiquitination and acetylation of the same residue (87) may regulate proteasomal degradation of Jhd1p. Functional studies into the ubiquitination of histone modifying enzymes in yeast are sparse; a single study has shown that polyubiquitination of Jhd2p by the E3 ubiquitin ligase, Not4p, promotes its turnover, leading to dysregulated H3K4 trimethylation levels and gene expression *in vivo* (31). Given the prevalence of crosstalk between phosphorylation and ubiquitination on mammalian histone MTases and DMases (28, 88, 89), it may be that closely positioned sites on yeast enzymes will positively or negatively influence one another and may jointly comprise a phosphodegron motif to control protein half-life.

This study homologously overexpressed histone methylation enzymes in *S. cerevisiae* in order to systematically identify PTM sites. A question is thus whether modifications found on these proteins represent those found on enzymes expressed at native levels. We note that there is a high concordance (61%) between phosphosites reported here and those identified previously in large-scale phosphoproteomic screens, which is strong evidence that our sites reflect those in natively expressed proteins. For our novel phosphorylation sites, many of these were likely not reported previously as they are either of low phosphorylation stoichiometry, making them difficult to detect in native phosphoproteomic analyses, or because they were found using proteases other than trypsin. We also note that there is a precedent for using overexpression to identify functionally important PTMs; our previous systematic analysis of overexpressed MTases (39) identified several phosphosites on Set5p that were subsequently shown to serve important regulatory roles on Set5p activity *in vivo* (30). Indeed, we have also shown here that PTMs that were confirmed on overexpressed Set2p regulate its function in cells.

In conclusion, this study represents the first systematic characterization of histone methylation enzymes and their PTMs in any eukaryote. This study also generated a draft regulatory network for these enzymes. The use of combinatorial mass spectrometry achieved near-complete sequence coverage of yeast histone MTases and DMases, and our targeted functional characterization of Set2p phosphosites revealed that phosphorylation can modulate enzyme activity *in vivo.* Taken together, these findings suggest that phosphorylation is an important “controller of the controllers.” Our detailed contextualization identified phosphosites that are invariant from yeast to human, of likely high functional importance, that should be prioritized for targeted analysis. Moving forward, mutagenesis of additional phosphosites will be useful to establish the effect of specific modifications on enzyme function, while kinase mapping will be essential to aid in the identification of upstream modifiers. Together, this will establish the exact way by which histone modifying enzymes, and their regulation of chromatin, interface with the signaling system of the cell.

## Experimental procedures

### Plasmids, mutagenesis, and yeast strains

Plasmids and primers used in this study are detailed in Tables S6 and S7, respectively. Overexpression plasmids encoding N-terminally hexahistidine-tagged *DOT1*, *JHD1*, *JHD2*, and *GIS1* from the Yeast GST Fusion Collection (Dharmacon, Lafayette, CO) (90) were a kind gift from Dr Gabriel Perrone. *SET5* with a C-terminal hexahistidine tag was cloned previously (39) into the Yeast ORF Collection BG1805 plasmid (Dharmacon). *SET2* and *RPH1* were amplified from *S. cerevisiae* genomic DNA and cloned into the pD1204 expression vector with C-terminal hexahistidine tags using the Electra Vector System (ATUM, Newark, CA) as per the manufacturer’s instructions, except that SapI (New England Biolabs, Ipswich, MA) endonuclease digestion was uncoupled from the T4 DNA ligase (New England Biolabs) reaction (91). All overexpression plasmids used in this study contain the galactose-inducible *GAL1* promoter and the *URA3* selectable marker for auxotrophic selection in yeast (Table S6). Resultant plasmid constructs were confirmed by Sanger sequencing using appropriate primers (Table S7) and transformed into wild-type *S. cerevisiae* haploid strain BY4741 *(MATa his3Δ1 leu2Δ0 met15Δ0 ura3Δ0)* using the lithium acetate method with *URA3* selection (92).

For generation of Set2p phosphomutant yeast strains, *SET2* was amplified from *S. cerevisiae* genomic DNA and cloned into the p426 shuttling vector using the Gibson Assembly cloning kit (New England Biolabs). Site-directed mutagenesis of p426-*SET2* was performed using relevant mutagenic oligonucleotides (Table S7) as described previously (93). *SET2* mutant-containing plasmids (Table S6) were verified by Sanger sequencing (see Table S7 for sequencing primers). Subsequently, mutated *SET2*, alongside the *URA3* selection cassette, was amplified and PCR products were transformed into *set2Δ* BY4741 yeast cells. Incorporation of desired mutations at the *SET2* chromosomal locus, by homologous recombination, was verified by Sanger sequencing (see Table S7 for sequencing primers).

### Yeast cell culture, recombinant protein expression, and affinity purification

Hexahistidine-tagged proteins were overexpressed in *S. cerevisiae* as done previously (39), with the exception that raffinose was not used as an intermediary carbon source to derepress the *GAL1* promoter. Instead, 150 ml yeast cultures in synthetic complete medium lacking uracil (SC-ura) supplemented with glucose (0.68% (w/v) yeast nitrogen base with ammonium sulfate (Sigma Aldrich, St Louis, MO), 0.4% (w/v) synthetic dropout medium without uracil (Sigma Aldrich), 2% (w/v) *D*-glucose) were grown at 30 °C and 200 rpm shaking to an optical density at 600 nm of 0.8. To alleviate repression of *GAL1* by glucose, cells were harvested by centrifugation, washed twice with sterile deionized water before resuspension in 150 ml SC-ura medium. Recombinant protein expression was then induced by the addition of 75 ml of 3× yeast extract peptone galactose medium (3% (w/v) yeast extract, 6% (w/v) bacteriological peptone, 6% (w/v) galactose) and carried out overnight at 30 °C and 200 rpm shaking. Following induction, yeast cells were harvested and stored at −80 °C until lysis and purification. Protein extraction and affinity purification of hexahistidine-tagged enzymes were performed as per (39), with the exception that yeast cell pellets were resuspended in 40 ml of hexahistidine-tag purification lysis buffer and that pan-phosphatase inhibitor (Roche, Basel, Switzerland) was added to purification buffers. Eluted proteins were concentrated and buffer-exchanged into phosphate-buffered saline (46.6 mM Na_2_HPO_4_, 3.4 mM NaH_2_PO_4_, 200 mM NaCl, pH 7.4) using Amicon Ultra-4 10K centrifugal filters (Merck Millipore, Burlington, MA) to a volume of 80 μl.

### SDS-PAGE and mass spectrometry sample preparation

Lysates (10 μl) and eluates (4 × 20 μl aliquots) were separated by SDS-PAGE and prepared for liquid chromatography mass spectrometry according to (94), with the addition of reduction and alkylation steps. This involved reducing gel bands with 10 mM dithiothreitol (DTT) at 37 °C for 1 h and their subsequent alkylation with iodoacetamide in the dark at ambient temperature for 1 h immediately before in-gel proteolytic digestion. Dried and rehydrated gel bands were incubated with trypsin (Promega, Fitchburg, WI, sequencing-grade, 50 ng in 20 mM NH_4_HCO_3_), LysargiNase (95) (Proteolysis Lab, IBMB-CSIC, 100 ng in 200 mM NH_4_HCO_3_, 10 mM CaCl_2_), Asp-N (Promega, sequencing-grade, 100 ng in 10 mM NH_4_HCO_3_), or chymotrypsin (Promega, sequencing-grade, 500 ng in 100 mM Tris-HCl (pH 8.0), 10 mM CaCl_2_) for 16 h in 100 μl reactions. Digests with trypsin, LysargiNase, and Asp-N were at 37 °C, while chymotryptic digestion was at 25 °C. Resultant peptides were extracted from gel bands and dried down in a SpeedVac (Thermo Fisher Scientific, Waltham, MA) before resuspension in 0.1% (v/v) formic acid, as described previously (94).

### Liquid chromatography mass spectrometry

Peptide samples were separated and ionized by nano-electrospray liquid chromatography using established methods (96) and then analyzed by high-resolution tandem mass spectrometry on an Orbitrap Fusion Lumos Tribrid Mass Spectrometer (Thermo Fisher Scientific) with data-dependent acquisition. Precursor scans were acquired in the orbitrap, and precursor ions were subsequently selected and isolated for fragmentation as described (97). Fragmentation using either higher-energy collisional dissociation (HCD) or electron transfer/higher-energy collisional dissociation (EThcD) was performed using standard instrument methods outlined in (97). HCD-MS/MS used a normalized collision energy of 30%, while EThcD-MS/MS used a supplemental activation collision energy of 28% for singly to triply charged fragment ions and no supplemental activation for higher charge state fragments. Fragment ions were detected in the Orbitrap (resolution = 30,000, scan range = 110–2000 *m/z*, maximum injection time = 125 ms for HCD, 250 ms for EThcD).

### Mass spectrometry data analysis

To identify putative modification sites on histone MTases and DMases, mass spectrometry data were analyzed on Proteome Discoverer 2.2 (Thermo Fisher Scientific) using basic PTM analysis processing and consensus workflows with primarily default settings (98). Data were searched against the SWISS-PROT (2019_06, 560,459 sequences) and contaminants (245 sequences) databases using the Mascot search engine (version 2.4, Matrix Sciences, London, United Kingdom). Search parameters were as follows: precursor and fragment ion mass tolerances were set to 5 ppm and 20 mmu, respectively; instrument was set to either ESI_HCD or EThcD depending on the fragmentation type; enzyme specificity was Trypsin, LysargiNase, Asp-N_ambic, or TrypChymo for respective preparations, with up to three missed cleavages permitted; dynamic modifications for all searches were carbamidomethyl (C), oxidation (M), and acetyl (peptide N-term), in addition to modifications exclusive to specific searches: phospho (STY) for phosphorylation; acetyl (K, protein N-term) for acetylation; methyl (K, DE), dimethyl (K) and trimethyl (K) for lysine methylation; methyl (R, DE) and dimethyl (R) for arginine methylation; GG (K) and RGG (K) for trypsin and LysargiNase ubiquitination searches, respectively; crotonyl (K) for crotonylation; ADP-ribosyl (C, D, E, K, N, R) for ADP-ribosylation; EQIGG (K) and REQIGG (K) for trypsin and LysargiNase SUMOylation searches, respectively. The ptm*RS* node was incorporated into processing workflows after database searching to provide localization probabilities for putative modification sites in the search output (ptm*RS* Best Site Probabilities column). False discovery rates (FDRs) for peptide-spectrum matches (PSMs) were determined by a decoy database search, and PSMs for modified peptides (FDR < 0.01) were filtered by ion score (≥30), expectation value (*p* < 0.05), and site localization probability (≥95%) and tabulated in Table S1. Fragmentation spectra for quality-filtered PSMs were manually inspected to verify unambiguous site localization and to monitor the abundance of diagnostic neutral loss fragments that may assist in the confident identification of certain PTMs. High-confidence modification sites that satisfied our stringent thresholding and spectral requirements are reported in this study.

For phosphopeptide abundance measurements, label-free quantification was performed as described previously (99). Briefly, the theoretical *m/z* of observed phosphorylated peptides, and their unmodified counterparts, were used to generate extracted ion chromatograms (XICs) in the Thermo Xcalibur Qual Browser 2.2, within a mass range window of 10 ppm. The ICIS algorithm was used to determine the area under the curve of XIC peaks, corresponding to either phosphorylated or unmodified versions of a peptide. The relative amount of phosphorylation present at a given site was then calculated by dividing the abundance of its cognate phosphopeptide by the total amount of peptide detected (phosphorylated + unmodified). Where phosphosites occur proximally to one another, isobaric phosphopeptides were differentially quantified by their unique chromatographic retention times. Specific details regarding phosphorylation quantification are summarized in Table S3.

### Contextualization and visualization of PTMs in sequence and structural features

Protein coverage maps were generated using the RStudio Desktop Package (version 1.2.5042-1, PBC, Boston, MA) from peptide sequences identified in database searches using an in-house script and visualized in GraphPad Prism (version 8.4.2, GraphPad Software Incorporated, San Diego, CA). Modification sites were mapped, to scale, onto linear protein representations using PTM VisQuant (Children’s Medical Research Institute, Sydney, Australia, https://visquant.cmri.org.au/). PTMs reported in the literature and on modification databases are summarized in Table S2 for phosphorylation, Table S4 for acetylation, and Table S5 for ubiquitination. Fragmentation spectra for exemplar PSMs were created using the Interactive Peptide Spectral Annotator tool (http://www.interactivepeptidespectralannotator.com/PeptideAnnotator.html, (100)), while disorder prediction was done using the DISOPRED3 algorithm on the PSIPRED online workbench (UCL Department of Computer Science: Bioinformatics Group, London, United Kingdom, http://bioinf.cs.ucl.ac.uk/psipred/). For three-dimensional contextualization of phosphosites, the following yeast protein structures were retrieved from the Protein Data Bank (RCSB): Dot1p (PDB ID: 1U2Z, (101)), Rph1p (PDB ID: 3OPW, (102)), and Set1p complexed with COMPASS subunits (Set1C; PDB ID: 6BX3, (77)). Structures were visualized in the PyMOL Molecular Graphics System (version 2.4.0, Schrödinger LLC, New York, NY) and colored according to figure legends.

### Sequence alignment

To establish the evolutionary conservation of histone MTase and DMase amino acid sequences, pairwise protein sequence alignments between all orthologous pairs of yeast and human histone methylation enzymes were performed on Geneious Prime (version 2020.1.2, Biomatters, Auckland, New Zealand) using a global alignment with free end gaps. For extended conservation analysis across Eukarya, sequences for relevant homologues of yeast enzymes in nematode (*Caenorhabditis elegans*), common fruit fly (*Drosophila melanogaster*), zebrafish (*Danio rerio*), African clawed frog (*Xenopus laevis*), and red junglefowl (*Gallus gallus*) were retrieved from UniProt (2020_04 release) and aligned by multiple sequence alignment in Geneious Prime using an identity cost matrix. Phosphorylation sites on mammalian enzymes were collated from the PhosphositePlus metadatabase (49) and a recent human phosphoproteome dataset (50).

### Network modeling

A draft regulatory network of histone methylation in *S. cerevisiae* was modeled using phosphoproteomic datasets, interaction databases, and prediction tools. NetworKIN3.0 (https://networkin.info/index.shtml, (52)) was used to predict upstream kinases (score > 2) that may phosphorylate histone MTase and DMase enzymes at observed phosphosites based on known kinase recognition sequences. With respect to phosphopeptide quantification, we consulted data from Bodenmiller *et al.* (53) and Holt *et al.* (54) to establish potential links between yeast protein kinases/phosphatases and specific phosphosites on histone methylation enzymes. Significant changes in phosphopeptide abundance in single gene deletion yeast strains (*p* < 0.05*;* fold-change < −1.5 for kinases, > 1.5 for phosphatases) were included in the regulatory network. Kinase–substrate interaction data from Ptacek *et al.* (55) and other low-throughput studies was also incorporated. Genetic interactions (*e.g.*, synthetic suppression, lethality) were collated from the Yeast Kinase Interaction Database (University of Toronto, Toronto, Canada, http://www.moseslab.csb.utoronto.ca/KID/, (56)).

### Quantification of H3K36 methylation by mass spectrometry

Wild-type and *SET2* mutant yeast strains were grown in triplicate 15 ml cultures to an optical density at 600 nm of 0.8 in yeast extract peptone dextrose medium (2% (w/v) *D*-glucose, 2% (w/v) bacteriological peptone, 1% (w/v) yeast extract). Cells were lysed in 300 μl HEPES lysis buffer (50 mM 4-(2-hydroxyethyl)-1-piperazineethanesolfonic acid (HEPES), 100 mM NaCl, 2 mM DTT, 2 mM EDTA, 0.5% (v/v) Triton-X-100, pH 7.5), supplemented with 1× protease inhibitor cocktail (Roche), by glass-bead homogenization, as done previously (39). Clarified lysates (30 μl) were electrophoresed on NuPAGE 12% Bis-Tris protein gels (Thermo Fisher) and gel bands corresponding to histone H3 prepared for mass spectrometric analysis as described above with the following modifications. In-gel derivatization of histones using propionic anhydride (Sigma Aldrich) was performed immediately prior to proteolytic digestion in order to generate uniform H3K36-containing tryptic peptides for relative quantification purposes, as described (103). Briefly, gel bands were rehydrated in 50 μl 50 mM NH_4_HCO_3_ and 100 μl propionic anhydride, vortexed, and incubated for 20 min at ambient temperature. Gel bands were then washed three times with 50 mM NH_4_HCO_3_, dried with 100% acetonitrile, and subject to an additional round of propionylation. Overnight in-gel tryptic digestion and peptide extraction were performed as above. Resultant peptides were analyzed by liquid chromatography MS/MS on an LTQ Orbitrap Velos Pro (Thermo Fisher) using collision-induced dissociation as per established methods (104), albeit without inclusion lists.

To quantify H3K36 methylation from mass spectrometry data, XICs were generated in the Thermo XCalibur Qual Browser 2.2 using the *m/z* of a triply-charged K36-containing tryptic peptide in its various methylation states. Given that propionylation of H3K36 can occur when it is un- or mono-methylated, but not when it is di^_^ or tri-methylated (105), the expected methylpeptides and their respective masses following in-gel derivatization are as follows: unmethylated, sequence K(propionyl)SAPSTGGV**K(propionyl)**K(propionyl)PHR, *m/z* = 539.97; monomethylated, sequence K(propionyl)SAPSTGGV**K(methyl+propionyl)**K(propionyl)PHR, *m/z* = 544.65; dimethylated, sequence K(propionyl)SAPSTGGV**K(dimethyl)**K(propionyl)PHR, *m/z* = 530.64; trimethylated, sequence K(propionyl)SAPSTGGV**K(trimethyl)**K(propionyl)PHR, *m/z* = 535.31. Label-free quantification was performed using the area under the curve of XIC peaks, as described above for phosphopeptide abundance measurement. The relative amount of methylation present at H3K36 was calculated by dividing the abundance of each methylation state by the total amount of peptide detected (unmodified + monomethylated + dimethylated + trimethylated).

For statistical analysis, the overall H3K36 methylation level for each *SET2* mutant was compared with the wild-type control using an ordinal logistic regression model with the four possible methylation states as response variables and the *SET2* mutation as a categorical covariate (Fig. S4*A*). This regression makes the proportional odds assumption, assuming that the odds of moving between adjacent categories (*i.e.*, methylation states) are equal. Using a likelihood ratio test, we found that fitting a more complex adjacent category model (Fig. S4*B*), which does not make the proportional odds assumption, explained significant variation in the data (*p* = 0.01). However, a comparison of the two regression models revealed that their respective outputs were not sensitive to the proportional odds assumption, and thus we have reported the common odds ratio and its statistical significance, per mutant, from the ordinal logistic model in Figure 7*B* for ease of interpretability.

## Data availability

All data described in this study are contained within the article and its supporting information, with the exception of the mass spectrometry proteomics data, which has been deposited to the ProteomeXchange Consortium *via* the PRIDE (106) partner repository with the data set identifier PXD021214.

## Conflict of interest

The authors declare that they have no conflicts of interest with the contents of this article.

## Supporting information

JBC_JBC/2020/015995_3

JBC_JBC/2020/015995_4
