# Supplementary material for: Post-translational modification analysis of Saccharomyces cerevisiae histone methylation enzymes reveals phosphorylation sites of regulatory potential
Source: J Biol Chem. 2020 Dec 20;296:100192. doi: 10.1074/jbc.RA120.015995 (PMC7948420; doi:10.1074/jbc.RA120.015995)
Supplement: JBC_JBC/2020/015995_3 [file mmc1.pdf]

Post-translational modification analysis of *Saccharomyces cerevisiae* histone methylation enzymes reveals phosphorylation sites of regulatory potential

Ryan J. Separovich, Mandy W.M. Wong, Tyler R. Chapman, Eve Slavich, Joshua J. Hamey, Marc R. Wilkins

Supporting information

Table S1: List of modified peptides identified on histone MTases and DMases

Table S2: Phosphorylation sites on *S. cerevisiae* histone MTase and DMase enzymes

Table S3: Label-free mass spectrometric quantification of phosphorylated peptides and their unmodified counterparts

Table S4: Acetylation sites on *S. cerevisiae* histone MTase and DMase enzymes

Table S5: Ubiquitination sites on *S. cerevisiae* histone MTase and DMase enzymes

Table S6: Plasmids used in this study

Table S7: Primers used in this study

Figure S1: Histidine-tag affinity purification or enrichment of yeast histone MTases and DMases

Figure S2: Annotated mass spectra of novel Dot1p phosphopeptides

Figure S3: Extracted ion chromatograms of different H3K36 methylation states in wild-type and *SET2* mutant yeast strains

Figure S4: Statistical comparison of H3K36 methylation levels across *SET2* mutants using ordinal regression and adjacent category models

Figure S5: Annotated mass spectra of novel Jhd1p ubiquitination sites and of 'pseudo' Set2p ubiquitination site

Figure S6: Examples of potential PTM crosstalk

Supplementary tables

Table S2: Phosphorylation sites on *S. cerevisiae* histone MTase and DMase enzymes

Enzyme	Phosphorylation site	Reference
**Set1p**	T504	(1)
	S625	(2)
	T653	(3)
	S655	(3)
	T659	(4)
	S701	(4)
	T704	(1)
	T705	(4)
	S708	(1)
	T785	(5)
	T787	(1)
	T875	(5)
	T1001	(1)
	T1006	(1)
**Set2p**	S6	This study, (6)
	S8	This study, (6)
	S10	This study, (6)
	T37	This study
	T127	(7)
	T429	This study
	S516	This study, (8)
	T518	(4)
	S522	This study, (5)
	S524	This study
	T531	This study
	S543	This study, (4)
	S572	(4)
	S585	(4)
	S718	This study, (4)
	S719	This study
	S726	(1)
	S730	(1)
**Set5p**	S13	This study, (9)
	S16	This study
	S26	(10)
	T90	(10)
	T94	(9)
	S96	(9)
	S134	(4)
	S281	This study
	S285	This study
	S288	This study
	T297	This study
	S301	This study, (10)
	T305	(10)
	S458	This study, (5)
	S461	This study, (11)
	S462	(9)
	S466	(9)
	S469	(9)
	T470	(9)

Enzyme	Phosphorylation site	Reference
**Set5p (*cont.*)**	S475	This study, (2)
	S476	This study, (2)
	T504	(10)
	T511	(9)
	S512	This study, (9)
	S517	This study, (11)
	S520	(10)
**Dot1p**	S14	This study
	S19	This study
	S24	This study
	S30	This study
	S44	(1)
	S46	(2)
	S74	This study
	T145	This study
	S174	This study
	S176	This study
	T208	This study
	S219	This study, (5)
	T223	(12)
	S227	This study
	S565	This study
	T576	This study, (13)
	Y580	(13)
**Jhd1p**	S44	This study
**Jhd2p**	S212	This study
	S266	This study, (4)
	S274	This study, (11)
	S340	This study
	S711	This study
**Rph1p**	S124	This study
	S139	(14)
	S378	(3)
	T381	This study, (3)
	T399	This study, (6)
	S405	(4)
	S410	(3)
	T411	(11)
	S412	This study, (15)
	S425	(13)
	S426	This study, (13)
	S429	This study, (16)
	S430	This study, (5)
	S434	(7)
	S458	(8)
	S459	This study, (15)
	T480	(6)
	S497	This study, (4)
	S501	This study, (4)
	S511	This study
	S552	(17)
	S555	(17)
	S557	This study, (15)
	S561	This study, (5)

Enzyme	Phosphorylation site	Reference
**Rph1p (*cont.*)**	S575	This study, (5)
	S584	This study, (6)
	S587	(18)
	S588	(4)
	S590	This study, (1)
	S626	(4)
	S652	This study, (15)
	T656	This study
	S659	This study, (3)
	S668	This study, (12)
	S688	This study, (11)
	S689	This study, (5)
**Gis1p**	T65	(8)
	S70	This study, (5)
	S109	(4)
	S115	(4)
	S132	(4)
	S343	This study, (15)
	S351	This study, (4)
	T353	(4)
	S365	(4)
	S373	(6)
	S374	(1)
	T405	This study, (5)
	S421	(12)
	S424	(8)
	S425	This study, (5)
	S580	(5)
	S670	This study, (4)
	S690	(6)
	S694	(15)
	S696	This study, (15)
	S734	This study, (6)
	S745	(11)
	S747	This study, (11)
	S838	(7)
	T844	(7)
	S858	(7)

Table S3: Label-free mass spectrometric quantification of phosphorylated peptides and their unmodified counterparts

Enzyme	Phosphosite	Experiment	Peptide sequence	Charge	*m/z* (Da)		Relative abundance (%)	
					Unmodified	Phospho	Unmodified	Phospho
**Set2p**	S6*	LysargiNase/EThcD	SKNQSVSASEDEKEILNNNAEGHKPQ	3+	965.79535	992.45079	99.23	0.77
	S8*	LysargiNase/EThcD	SKNQSVSASEDEKEILNNNAEGHKPQ	3+	965.79535	992.45079	92.49	7.51
	S10*	LysargiNase/EThcD	SKNQSVSASEDEKEILNNNAEGHKPQ	3+	965.79535	992.45079	87.37	12.63
	S516	Trypsin/HCD	SNTQVNSPSSSGIPK	2+	751.8759	791.85907	98.28	1.72
	S522	Asp-N/HCD	SNTQVNSPSSSGIPKTPGALDSK	3+	758.05203	784.70748	91.31	8.69
	S524*	Trypsin/HCD	SNTQVNSPSSSGIPKTPGALDSK	3+	758.05203	784.70748	94.84	5.16
	T531*	Trypsin/HCD	SNTQVNSPSSSGIPKTPGALDSK	3+	758.05203	784.70748	96.98	3.02
	S543	Trypsin/HCD	KHKLSDEEYER	2+	717.3546	757.33777	96.76	3.24
	S718	Trypsin/HCD	KALALSSASTR	2+	552.82221	592.80538	99.51	0.49
	S719	LysargiNase/HCD	KKALALSSAST	2+	538.81914	578.8023	99.80	0.20
						AVERAGE:	***95.66***	***4.34***
**Set5p**	S13*	LysargiNase/HCD	KIGTLNDSDQSAVHNGTENGSDF	2+	1203.54384	1243.527	99.90	0.10
	S16*	LysargiNase/HCD	KIGTLNDSDQSAVHNGTENGSDF	2+	1203.54384	1243.527	99.96	0.04
	S281	Chymo/EThcD	IKLRDASGIGSTF	3+	455.58926	482.24471	99.83	0.17
	S285	Asp-N/HCD	DASGIGSTFSLLNGTTVHTEEES	2+	1176.54552	1216.52868	99.94	0.06
	S288	Asp-N/HCD	DASGIGSTFSLLNGTTVHTEEES	2+	1176.54552	1216.52868	99.91	0.09
	T297*	LysargiNase/HCD	RDASGIGSTFSLLNGTTVHTEEESDNGTK	3+	1008.4778	1035.13324	99.81	0.19
	S301*	LysargiNase/HCD	RDASGIGSTFSLLNGTTVHTEEESDNGTK	3+	1008.4778	1035.13324	99.45	0.55
	S458*	Trypsin/HCD	NADANLGVEKIDSNDSSEDGSKK	2+	798.37361	825.02905	88.59	11.41
	S461*	Trypsin/HCD	NADANLGVEKIDSNDSSEDGSKK	2+	798.37361	825.02905	89.66	10.34
	S475	Trypsin/HCD	KSSMREAQPDLKEILK	4+	473.00948	493.00106	96.74	3.26
	S476	Asp-N/EThcD	DSSEDGSKKSTGNRKSSMREAQP	3+	828.05774	854.71318	96.37	3.63
	S512	Asp-N/EThcD	DTQGNVRKTSVRF	3+	503.27071	529.92615	99.69	0.31
	S517	LysargiNase/HCD	RFDSNVSVAVDE	2+	669.32023	709.3034	99.02	0.98
						AVERAGE:	***97.61***	***2.39***
**Dot1p**	S14	Asp-N/HCD	DSFIMSSPNL	2+	563.75789	603.74105	99.58	0.42
	S19*	Chymo/HCD	IMSSPNLDSQESSISPIDEK	2+	1097.01501	1136.99817	90.23	9.77
	S30*	Chymo/HCD	IMSSPNLDSQESSISPIDEK	2+	1097.01501	1136.99817	89.41	10.59
	S74	LysargiNase/HCD	KQVQNLLEEANKYDPIYGSSLPRGFL	3+	993.85733	1020.51278	98.64	1.36
	T145	Trypsin/EThcD	TNHKHTPISKQEIDTAR	3+	659.34846	686.0039	99.79	0.21
	S174	Trypsin/EThcD	ANKKNDRDSPSSTFVDWNGPCLR	3+	888.75763	915.41307	97.79	2.21
	S176	Trypsin/EThcD	KNDRDSPSSTFVDWNGPCLR	3+	784.36596	811.0214	98.53	1.47
	T208	Trypsin/EThcD	SHEIYSGTPIQSISLR	3+	596.64773	623.30317	99.47	0.53
	S219	Trypsin/EThcD	TNSPQPTSLTSDNDTSSVTTAK	3+	751.35567	778.01111	88.86	11.14
	S227	Trypsin/HCD	TNSPQPTSLTSDNDTSSVTTAK	3+	751.35567	778.01111	99.11	0.89
	T576	Trypsin/HCD	RNRGTPVKYTR	3+	449.92498	476.58042	90.48	9.52
						AVERAGE:	***95.63***	***4.37***

Enzyme	Phosphosite	Experiment	Peptide sequence	Charge	*m/z* (Da)		Relative abundance (%)	
					Unmodified	Phospho	Unmodified	Phospho
**Jhd1p**	S44	Trypsin/HCD	IHYSNLTSSEVLSYPNSAK	2+	1055.5262	1095.50937	97.90	2.10
**Jhd2p**	S212	Asp-N/HCD	DQYPKSLLSDDE	2+	705.32518	745.30834	94.22	5.78
	S266	Trypsin/HCD	TILCDSCDKPFHIYCLSPPLER	3+	907.76596	934.42141	96.66	3.34
	S274	Asp-N/HCD	DKPFHIYCLSPPLERVPSG	3+	738.0455	764.70094	79.34	20.66
	S340	Trypsin/HCD	RSSLTTVKYGADIHNELPGQITGFPTR	3+	986.85258	1013.50803	99.27	0.73
	S711	Asp-N/HCD	DELYFTKSLK	2+	622.33208	662.31524	99.76	0.24
						AVERAGE:	***93.85***	***6.15***
**Rph1p**	T381	LysargiNase/EThcD	KGTPPLNQLPNPAMPLLH	3+	646.69251	673.34795	98.90	1.10
	T399	Chymo/EThcD	LHRPTLKEMESSSL	3+	548.61889	575.27434	98.12	1.88
	S412	Trypsin/EThcD	STSPDVGHFSNFK	2+	711.83605	751.81921	84.80	15.20
	S426	Trypsin/HCD	SKSSGVSSPLLSR	2+	652.86207	692.84523	94.46	5.54
	S429*	Trypsin/EThcD	SSGVSSPLLSR	2+	545.29857	585.28174	99.98	0.02
	S430*	Trypsin/EThcD	SSGVSSPLLSR	2+	545.29857	585.28174	91.64	8.36
	S459	Trypsin/HCD	ISSFQEQPLNK	2+	645.83806	685.82122	86.30	13.70
	S497	LysargiNase/HCD	RETSQTAMLTDHEDNIVAMSLTSMANSAASSP	3+	1138.84131	1165.49676	97.46	2.54
	S501	Chymo/HCD	TSMANSAASSPRLPL	2+	759.88267	799.86584	94.55	5.45
	S511	Chymo/EThcD	SRLNSSNEL	2+	510.25944	550.24261	87.15	12.85
	S557	LysargiNase/EThcD	KNISGISHSAPHSPVNPNISLIK	3+	804.11156	830.767	88.19	11.81
	S561	LysargiNase/EThcD	KNISGISHSAPHSPVNPNISLI	3+	761.41324	788.06868	74.08	25.92
	S575	Trypsin/EThcD	VKSPNIVTLNISR	2+	720.93028	760.91345	81.31	18.69
	S590	Trypsin/EThcD	ESSRSPIALNYEAR	3+	531.60575	558.2612	81.81	18.19
	S652	Trypsin/HCD	ESPVETSKSNLILSK	2+	816.44616	856.42932	98.73	1.27
	S659	Trypsin/EThcD	ESPVETSKSNLILSK	3+	544.6332	571.28864	99.47	0.53
	S668	Chymo/HCD	SKVASTRQEDSF	2+	677.83351	717.81667	88.95	11.05
	S688	LysargiNase/HCD	RNDDLDKEQGSSPLNS	3+	592.27825	618.9337	92.43	7.57
	S689	Trypsin/HCD	NDDLDKEQGSSPLNSK	3+	582.94287	609.59832	89.45	10.55
						AVERAGE:	***90.90***	***9.10***
**Gis1p**	S70	Trypsin/HCD	TIQLDSPIQQQAK	2+	735.40156	775.38472	98.27	1.73
	S343	Asp-N/HCD	DSNESEQRGSITDNDN	2+	890.86445	930.84761	98.84	1.16
	S351	LysargiNase/HCD	RGSITDNDNDLFQKV	2+	861.42629	901.40945	90.57	9.43
	T405	Trypsin/HCD	STTPNGVNQFLNMNQTTISR	3+	747.03057	773.68602	93.10	6.90
	S425	Trypsin/HCD	ISSPLLSR	2+	436.76363	476.7468	90.87	9.13
	S696	LysargiNase/EThcD	KSPISSFVNDY	2+	628.81151	668.79468	94.70	5.30
	S734*	LysargiNase/HCD	RQNSNNINPLDAGPSFSPLH	3+	726.6944	753.34985	96.53	3.47
	S747*	LysargiNase/HCD	RQNSNNINPLDAGPSFSPLH	3+	726.6944	753.34985	89.69	10.31
						AVERAGE:	***94.07***	***5.93***

**** isobaric phosphopeptides in adjacent rows were differentially quantified by their unique chromatographic retention times***

Table S4: Acetylation sites on *S. cerevisiae* histone MTase and DMase enzymes

Enzyme	Acetylation site	Reference
**Set1p**	K982	(19)
**Set2p**	K3	This study
	K14	This study
	K43	This study
	K55	This study
	K116	This study
	K117	This study
	K126	This study
	K128	This study
	K172	This study
	K215	This study
	K225	This study
	K228	This study
	K329	This study
	K340	This study
	K412	This study
	K428	This study
	K433	This study
	K447	This study
	K450	This study
	K459	This study
	K500	This study
	K510	This study
	K530	This study
	K541	This study
	K566	This study
	K574	This study
	K578	This study
	K584	This study
	K598	This study
	K602	This study
	K607	This study
	K620	This study
	K667	This study
	K672	This study
	K675	This study
	K695	This study
	K713	This study
**Set5p**	K6	This study
	K30	This study
	K99	This study
	K101	This study
	K113	This study
	K130	This study
	K135	This study
	K141	This study
	K144	This study
	K154	This study
	K169	This study
	K178	This study
	K197	This study
	K200	This study
	K265	This study

Enzyme	Acetylation site	Reference
**Set5p (*cont.*)**	K276	This study
	K306	This study
	K390	This study
	K444	This study
	K445	This study
	K455	This study
	K467	This study
	K485	This study
**Dot1p**	K50	This study
	K87	This study
	K89	This study
	K99	(19)
	K143	This study
	K158	This study
	K168	This study
	K238	This study
	K289	This study
	K336	This study, (19)
	K443	This study
**Jhd1p**	K91	This study
	K437	This study
**Jhd2p**	K70	This study
	K93	This study
	K119	This study
	K130	This study
	K147	This study
	K258	This study
	K345	This study
	K504	This study
	K659	(7)
	K602	This study
	K659	This study
	K676	This study
	K687	This study
	K720	This study
	K725	This study
**Rph1p**	K3	This study
	K214	This study
	K288	This study
	K424	This study
	K665	(20)
	K684	(20)
	K694	(20)
	K705	This study
**Gis1p**	K81	This study
	K448	(20)
	K680	(20)
	K813	(20)

Table S5: Ubiquitination sites on *S. cerevisiae* histone MTase and DMase enzymes

Enzyme	Ubiquitination site	Reference
**Set1p**	K636	(21)
	K637	(21)
**Dot1p**	K43	(12)
	K50	(12)
	K238	(12)
**Jhd1p**	K91	This study
	K122	This study

Table S6: Plasmids used in this study

Plasmid	Relevant markers	Source
pEGH-*DOT1*	amp^R and *URA3* selection markers<br>*GAL1* promoter<br>N-terminal hexahistidine and glutathione *S*-transferase (GST) tags	Yeast GST Fusion Collection (Gabriel Perrone)
pEGH-*JHD1*		
pEGH-*JHD2*		
pEGH-*GIS1*		
BG1805-*SET5*	amp^R and *URA3* selection markers<br>*GAL1* promoter<br>C-terminal hexahistidine tag	Yeast ORF Collection (Daniel Winter)
pD1204-*SET2*	amp^R and *URA3* selection markers<br>*GAL1* promoter<br>C-terminal hexahistidine tag	This study
pD1204-*RPH1*		
p426-*SET2*	amp^R and *URA3* selection markers	This study
p426-*SET2*-S6A		
p426-*SET2*-S6D		
p426-*SET2*-S8A		
p426-*SET2*-S8D		
p426-*SET2*-S10A		
p426-*SET2*-S10D		
p426-*SET2*-S6/8/10A		
p426-*SET2*-S6/8/10D		

Table S7: Primers used in this study

Primer name	Sequence (5’→ 3’)*	Purpose
SET2-Electra-Fwd	tacacgtacttagtcgctgaagctcttctATGTCGAAGAACCAAAGT	Electra cloning of *SET2* and *RPH1* into pD1204 expression vector
SET2-Electra-Rev	aggtacgaactcgattgacggctcttctaccttattagtgatggtgatggtgatgTGATGATG TTGAAGG	
RPH1-Electra-Fwd	tacacgtacttagtcgctgaagctcttctATGACGAAACTAATCGCT	
RPH1-Electra-Rev	aggtacgaactcgattgacggctcttctaccttattagtgatggtgatggtgatgGTTTAAAG GTGTACT	
pD1204-check-Fwd	CTTCTCAAGCAAGGTTTTCAG	Confirmation sequencing of pD1204 plasmids
pD1204-check-Rev	ATTGGCAGTAACCTGGCC	
p426-Fwd173	GAGAGTGCACCATACCACAGC	Gibson assembly cloning of *SET2* into p426 shuttle vector
p426-Rev145	CTGCTCTGATGCCGCATAGT	
SET2-Gibson-Fwd	actatgcggcatcagagcagATGTCGAAGAACCAAAGTGTGAGTGCG TCGGAAGATG	
SET2-Gibson-Rev	gctgtggtatggtgcactctcTTATGATGATGTTGAAGGTGGAGGA	
p426-check-Fwd	CTTGTCTGTAAGCGGATGC	Confirmation sequencing of p426-*SET2*
p426-check-Rev	CACCGCATAGGGTAATAACTG	
SET2-S6A-Fwd	TCGAAGAACCAAgctGTGAGTGCGTCG	Site-directed mutagenesis of p426-*SET2*
SET2-S6A-Rev	CGACGCACTCACAgcTTGGTTCTTCGA	
SET2-S6D-Fwd	TCGAAGAACCAAgacGTGAGTGCGTCG	
SET2-S6D-Rev	CGACGCACTCACgtcTTGGTTCTTCGA	
SET2-S8A-Fwd	AACCAAAGTGTGgcTGCGTCGGAAGAT	
SET2-S8A-Rev	ATCTTCCGACGCAgcCACACTTTGGTT	
SET2-S8D-Fwd	AACCAAAGTGTGgacGCGTCGGAAGAT	
SET2-S8D-Rev	ATCTTCCGACGCgtcCACACTTTGGTT	
SET2-S10A-Fwd	AGTGTGAGTGCGgcgGAAGATGAAAAA	
SET2-S10A-Rev	TTTTTCATCTTCcgcCGCACTCACACT	
SET2-S10D-Fwd	AGTGTGAGTGCGgacGAAGATGAAAAA	
SET2-S10D-Rev	TTTTTCATCTTCgtcCGCACTCACACT	
SET2-S6-8-10A-Fwd	TCGAAGAACCAAgccGTGgccGCGgCcGAAGATGAAAAA	
SET2-S6-8-10A-Rev	TTTTTCATCTTCgGcCGCggcCACggcTTGGTTCTTCGA	
SET2-S6-8-10D-Fwd	TCGAAGAACCAAgacGTGgacGCGgacGAAGATGAAAAA	

Primer name	Sequence (5’→ 3’)*	Purpose
SET2-S6-8-10D-Rev	TTTTTCATCTTCgtcCGCgtcCACgtcTTGGTTCTTCGA	Site-directed mutagenesis of p426-*SET2*
SET2-genomic-Fwd	tagtcgtgctgtcaaacctttctcctttcctggttgttgttttacgtgatcATGTCGAAGAACCAAAGTGTGAG	Amplification of *SET2-URA3* for homologous recombination
SET2-S6A-genomic-Fwd	tagtcgtgctgtcaaacctttctcctttcctggttgttgttttacgtgatcATGTCGAAGAACCAAgcTG	
SET2-S6D-genomic-Fwd	tagtcgtgctgtcaaacctttctcctttcctggttgttgttttacgtgatcATGTCGAAGAACCAAgacGTG	
SET2-S6-8-10A-genomic-Fwd	tagtcgtgctgtcaaacctttctcctttcctggttgttgttttacgtgatcATGTCGAAGAACCAAgcTG	
SET2-S6-8-10D-genomic-Fwd	tagtcgtgctgtcaaacctttctcctttcctggttgttgttttacgtgatcATGTCGAAGAACCAAgacGTG	
SET2-URA3-genomic-Rev	ctttgggacagaaaacgtgaaacaagccccaaatatgcatgtctggttaaTTAGTTTTGCTGGCCGCA	
SET2-A-Fwd	GAGAAGAAGCTGACTTCGACTATTG	Verify integration of *URA3* at *SET2* locus
SET2-D-Rev	AAAAATAAAGACACTTGAAACGCAC	
SET2-seq-Fwd	GTGTGATAGAAAACTGCATAG	Confirmation sequencing of chromosomal *SET2*
SET2-mid-seq-Fwd	AAGACTCAAACAGATGCGGC	
URA3-seq-Rev	CGTGCTGCTACTCATCCTAG	

***** Non-annealing nucleotides are shown in lowercase***

Supplementary figures

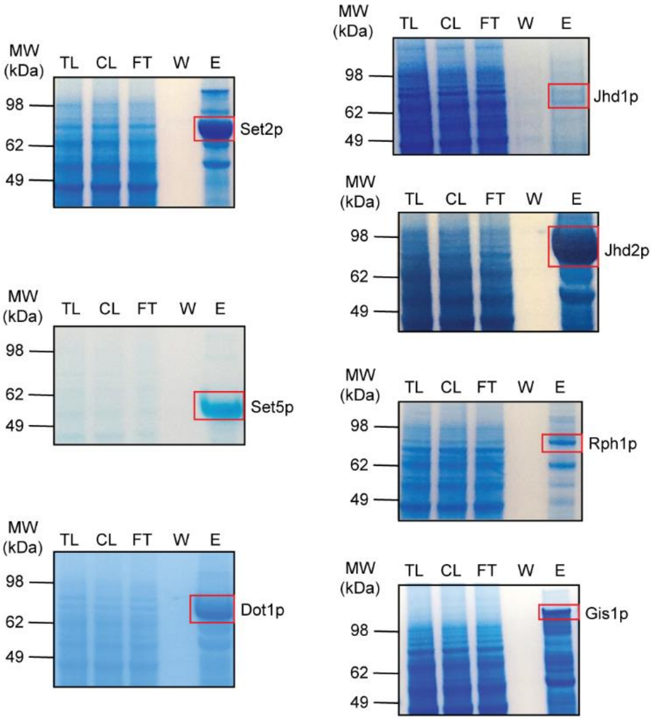

Figure S1: Histidine-tag affinity purification or enrichment of yeast histone MTases and DMases. Polyacrylamide gel electrophoresis (PAGE) confirms successful purification and/or enrichment of homologously overexpressed and hexahistidine-tagged histone MTase (left) and DMase (right) enzymes from *S. cerevisiae*. Fractions were collected throughout the purification and electrophoresed: total *S. cerevisiae* (BY4741) lysate (TL), clarified lysate (CL), flow-through (FT), wash (W), and purified eluate (E). The expected sizes for each tagged enzyme is as follows: Set2p = 84 kDa (left, top); Set5p = 61 kDa (left, middle); Dot1p = 92 kDa (left, bottom); Jhd1p = 82 kDa (right, top); Jhd2p = 110 kDa (right, second top); Rph1p = 90 kDa (right, second bottom); Gis1p = 125 kDa (right, bottom).

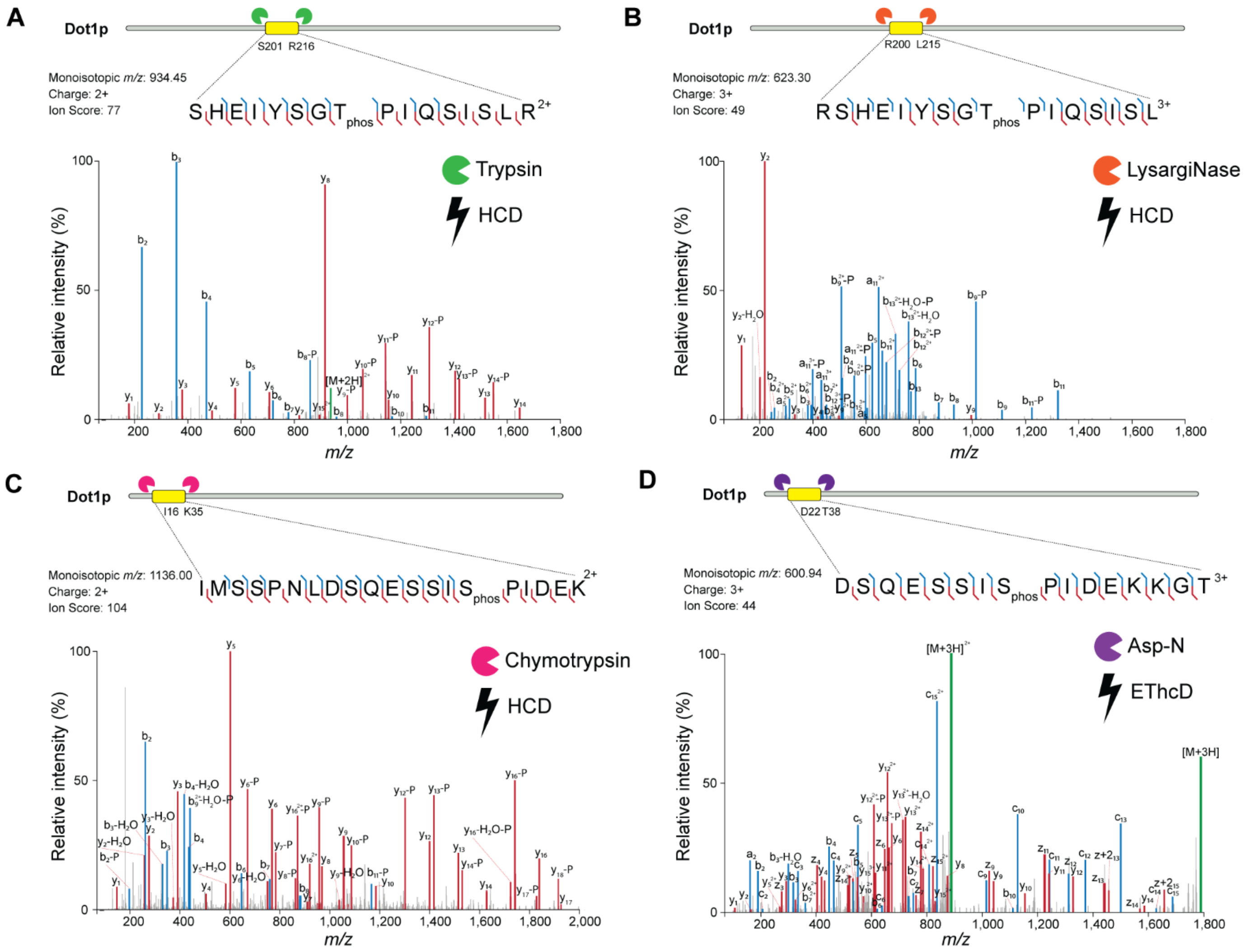

Figure S2: Annotated mass spectra of novel Dot1p phosphopeptides. Observed N-terminal and C-terminal fragment ions are shown in blue and red, respectively, while precursor ions are shown in green. Fragment ions with a neutral loss of water and/or phosphoric acid are denoted by - H_2O and - P. The proteolytic digestion and fragmentation method used to generate each MS/MS spectrum is shown (middle right of all panels). Fragmentation maps above their corresponding spectra illustrate the unambiguous identification of two exemplar Dot1p phosphorylation sites, not observed previously. (A) HCD fragmentation spectrum for a doubly charged tryptic Dot1p phosphopeptide SHEYSGT$_{phos}$PIQSISLR (*m/z* = 934.45, score = 77) identified phosphorylation of threonine 208. (B) HCD fragmentation spectrum for a triply charged lysargiNase-generated Dot1p phosphopeptide RSHEYSGT$_{phos}$PIQSISL (*m/z* = 623.30, score = 49) independently verified threonine 208 phosphorylation. (C) HCD fragmentation spectrum for a doubly charged chymotryptic Dot1p phosphopeptide IMSSPNLDSQESSIS$_{phos}$PIDEK (*m/z* = 1136.00, score = 104) identified phosphorylation of serine 30. (D) EThcD fragmentation spectrum for a triply charged Asp-N generated Dot1p phosphopeptide DSQESSIS$_{phos}$PIDEKKGT (*m/z* = 600.94, score = 44) independently verified serine 30 phosphorylation.

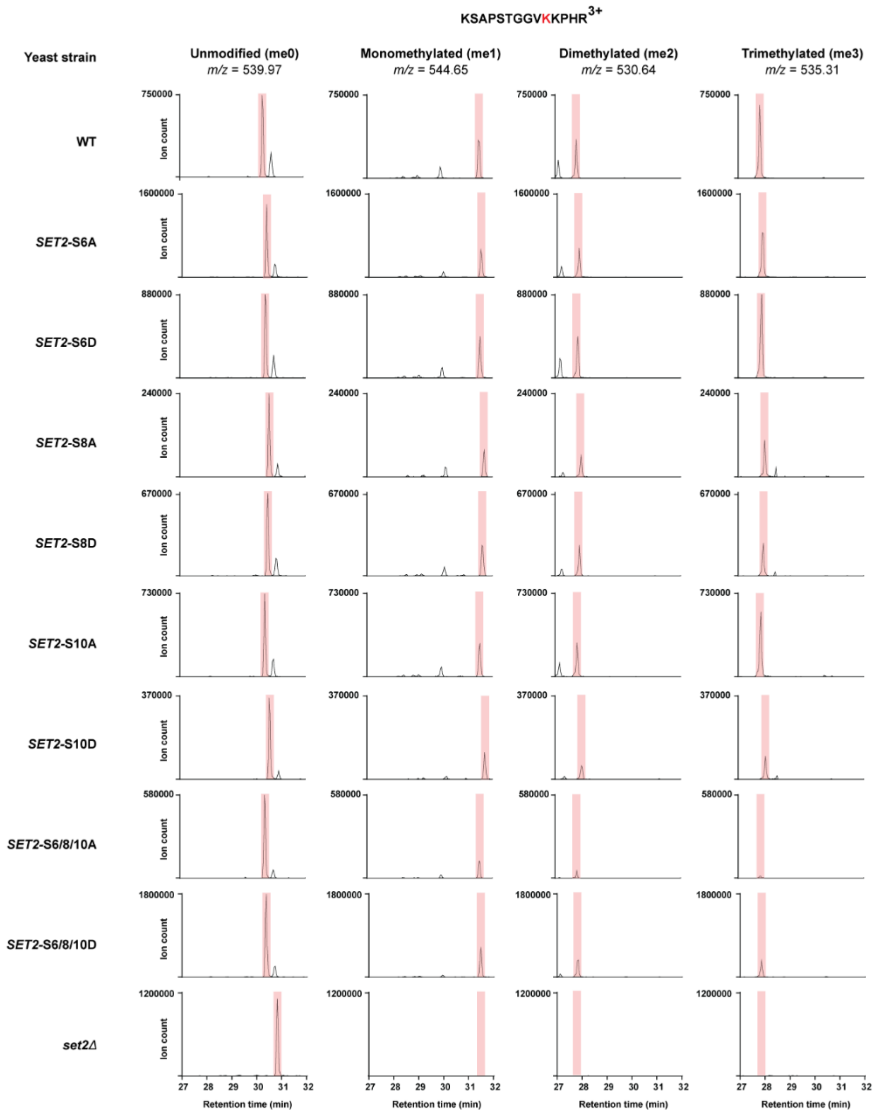

Figure S3: Extracted ion chromatograms (XICs) of different H3K36 methylation states in wild-type and *SET2* mutant yeast strains. XICs were generated in the Thermo Xcalibur Qual Browser 2.2 using the *m/z* of a triply-charged, K36-containing tryptic peptide in its unmodified (sequence K(propionyl)SAPSTGGV**K(propionyl)**K(propionyl)PHR; *m/z* = 539.97), monomethylated (sequence K(propionyl)SAPSTGGV**K(methyl+propionyl)**K(propionyl)PHR; *m/z* = 544.65), dimethylated (sequence K(propionyl)SAPSTGGV**K(dimethyl)**K(propionyl)PHR; *m/z* = 530.64), and trimethylated (sequence K(propionyl)SAPSTGGV**K(trimethyl)**K(propionyl)PHR; *m/z* = 535.31) forms. Total ion counts were normalised to the most abundant methylation state detected for each mutant. Chromatographic peaks corresponding to the unmodified, mono-, di-, and tri-methylated forms of H3K36 are shaded in coral rectangles. Areas under the curve of shaded peaks were used to quantify the *in vivo* distribution of H3K36 methylation, and represent a single biological replicate included in Figure 7B.

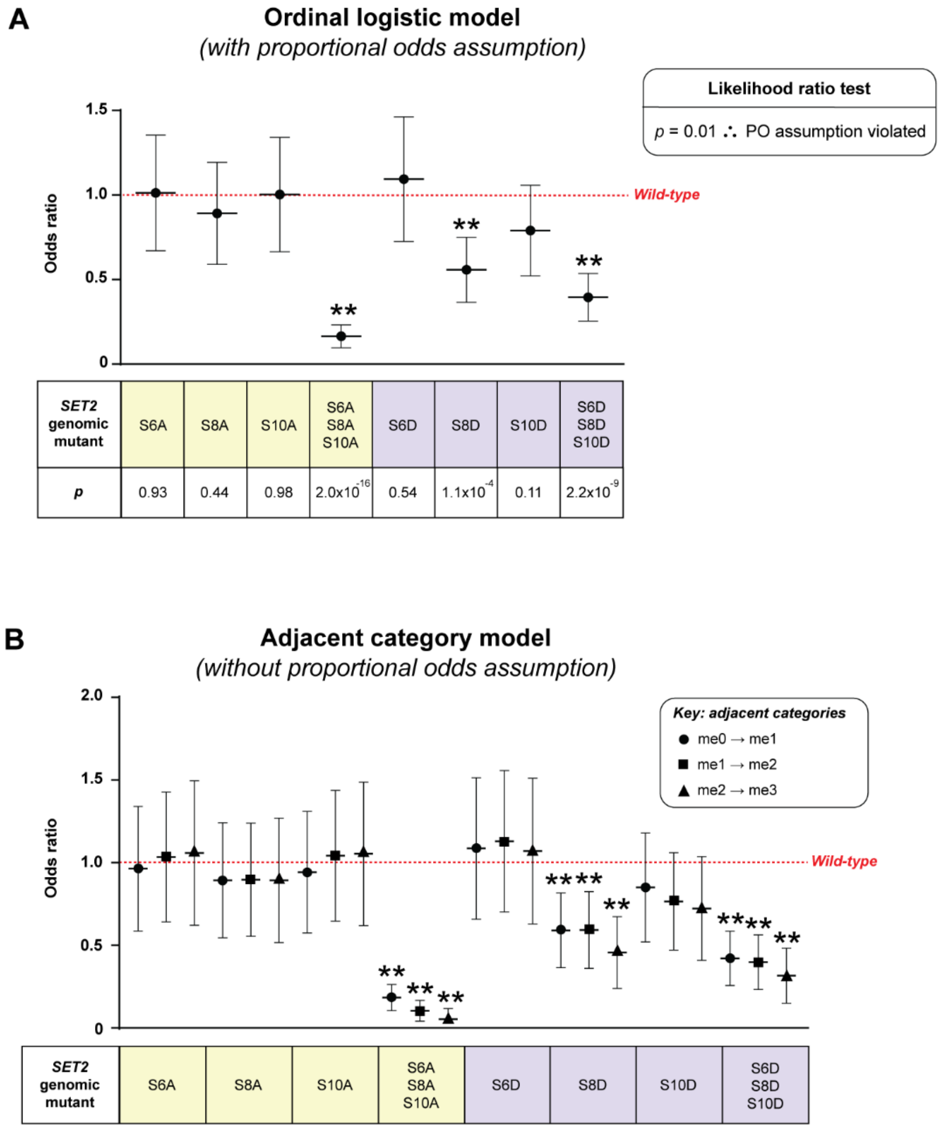

Figure S4: Statistical comparison of H3K36 methylation levels across *SET2* mutants using ordinal regression (A) and adjacent category (B) models. For each mutant strain, the odds ratio of H3K36 being more methylated than the wild-type control (normalised to 1.0; dotted red line) was averaged across $n = 3$ biological replicates, with error bars denoting a 95% confidence interval (**, $p < 0.01$ versus WT). (A) Ordinal logistic regression modelling of overall changes in H3K36 methylation levels. This regression assumes that the odds of moving between adjacent categories (i.e. methylation states) are equal. For each mutant strain, the statistical significance of alterations in methylation compared to the wild-type control are tabulated as *p*-values. The validity of the proportional odds (PO) assumption was checked with a likelihood ratio test. (B) Adjacent category modelling of changes between specific H3K36 methylation states. This model does not assume proportional odds, and fits a separate odds ratio for each pair of adjacent states. The odds ratio of each mutant having H3K36 in a higher adjacent methylation state, with respect to wild-type, is plotted for each pairwise comparison as either circles (unmethylated (me0) to monomethylated (me1)), squares (me1 to dimethylated (me2)), or triangles (me2 to trimethylated (me3)). Outputs from this model are not qualitatively different to those obtained using an ordinal regression approach (A). Thus, we have reported the common odds ratio (per mutant) and its statistical significance from the ordinal logistic model in Fig. 7B.

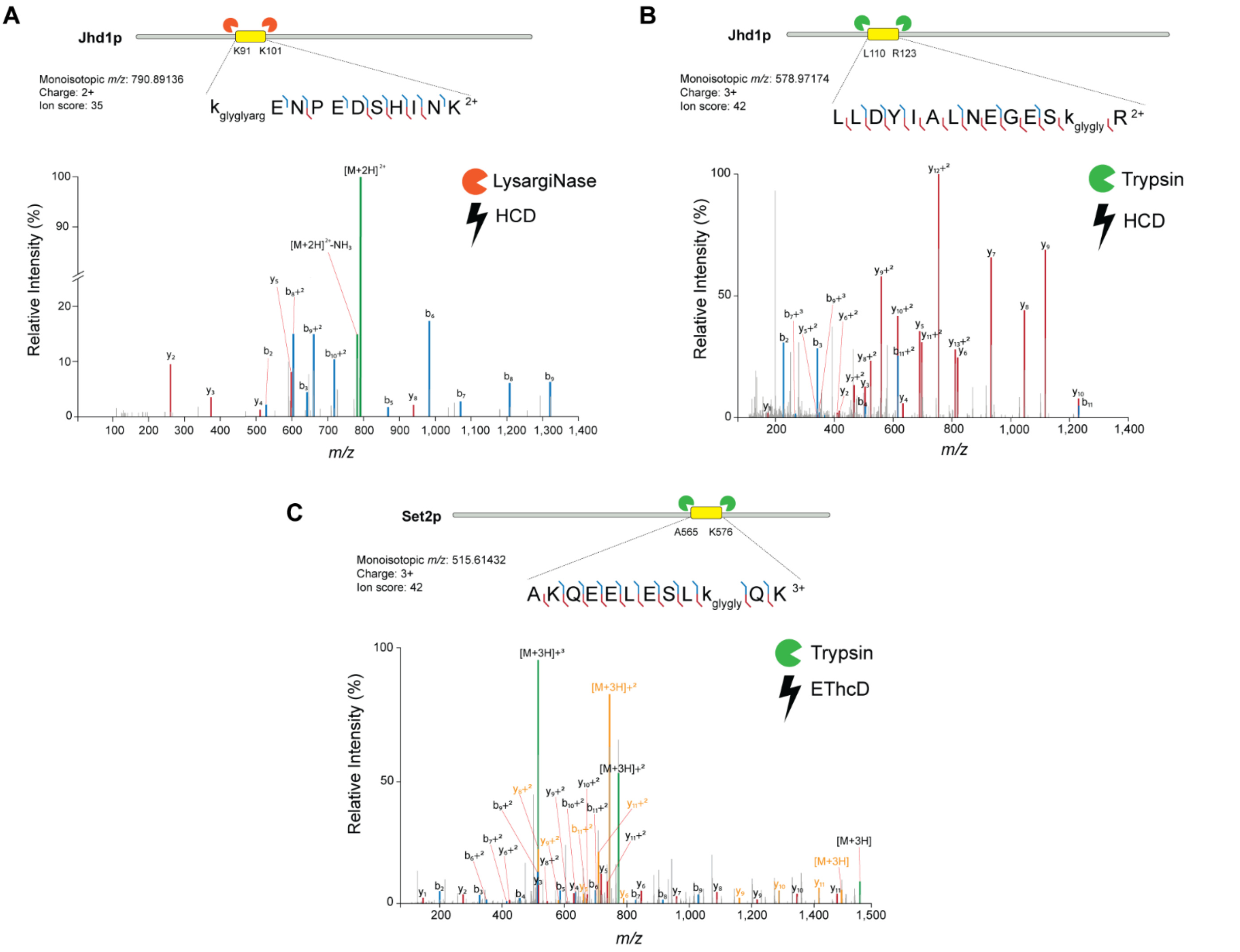

Figure S5: Annotated mass spectra of novel Jhd1p ubiquitination sites and of 'pseudo' Set2p ubiquitination site. Observed N-terminal and C-terminal fragment ions are shown in blue and red, respectively, while precursor ions are shown in green. Ions with a neutral loss of ammonia are denoted by -NH3. The proteolytic digestion and fragmentation method used to generate each MS/MS spectrum is shown (middle right of all panels). Fragmentation maps above their corresponding spectra illustrate the identification of two novel Jhd1p ubiquitination sites. (A) HCD fragmentation spectrum for a double charged, LysargiNase-generated Jhd1p ubiquitinated peptide $K_{glyglyarg}$ENPEDSHINK (*m/z* = 790.89136, score = 35) identified ubiquitination of lysine 91. (B) HCD fragmentation spectrum for a double charged tryptic Jhd1p ubiquitinated peptide LLDYIALNEGESK$_{glygly}$R (*m/z* = 578.97174, score = 42) identified ubiquitination of lysine 122. (C) EThcD fragmentation spectrum for triply charged, tryptic peptide AKQEELESLK$_{glygly}$QK (*m/z* = 515.61432, score = 42) initially identifies lysine 574 as a ubiquitination site. However, the presence of 57 Da neutral losses, peaks shown in yellow, reveal this as a 'pseudo' ubiquitination site.

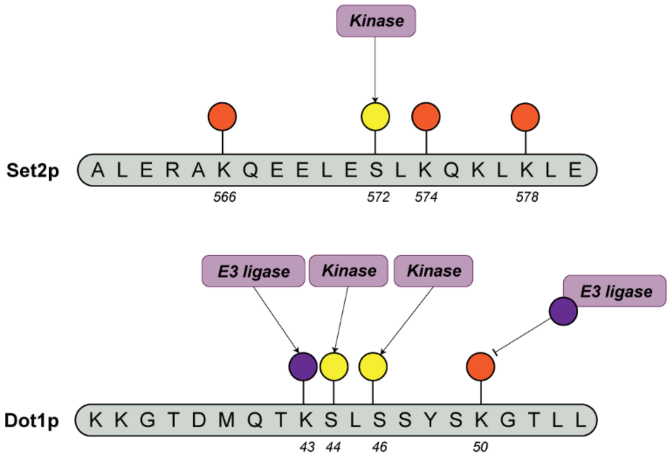

Figure S6: Examples of potential PTM crosstalk. Two examples of closely positioned PTMs on Set2p and Dot1p which have the potential to crosstalk with one another. Phosphorylation (yellow), acetylation (orange), and ubiquitination (purple) sites are illustrated on linear sequence maps, with the upstream regulators of unknown identity coloured in mauve. It is possible and likely that these clustered PTMs will affect, either positively or negatively, the subsequent deposition of a modification at a nearby residue.

Supplementary references

1. Li, J., Paulo, J. A., Nusinow, D. P., Huttlin, E. L., and Gygi, S. P. (2019) Investigation of Proteomic and Phosphoproteomic Responses to Signaling Network Perturbations Reveals Functional Pathway Organizations in Yeast. *Cell Rep.* **29**, 2092-2104. e2094
2. Chi, A., Huttenhower, C., Geer, L. Y., Coon, J. J., Syka, J. E., Bai, D. L., Shabanowitz, J., Burke, D. J., Troyanskaya, O. G., and Hunt, D. F. (2007) Analysis of phosphorylation sites on proteins from Saccharomyces cerevisiae by electron transfer dissociation (ETD) mass spectrometry. *P. Natl. Acad. Sci. U.S.A.* **104**, 2193-2198
3. Iesmantavicius, V., Weinert, B. T., and Choudhary, C. (2014) Convergence of ubiquitylation and phosphorylation signaling in rapamycin-treated yeast cells. *Mol. Cell Proteomics* **13**, 1979-1992
4. [preprint] Lanz, M. C., Yugandhar, K., Gupta, S., Sanford, E., Faça, V., Vega, S., Joiner, A., Fromme, C., Yu, H., and Smolka, M. B. (2019) In-depth and 3-Dimensional Exploration of the Budding Yeast Phosphoproteome. *bioRxiv*, 10.1101/700070
5. Albuquerque, C. P., Smolka, M. B., Payne, S. H., Bafna, V., Eng, J., and Zhou, H. (2008) A multidimensional chromatography technology for in-depth phosphoproteome analysis. *Mol. Cell Proteomics* **7**, 1389-1396
6. Holt, L. J., Tuch, B. B., Villén, J., Johnson, A. D., Gygi, S. P., and Morgan, D. O. (2009) Global analysis of Cdk1 substrate phosphorylation sites provides insights into evolution. *Science* **325**, 1682-1686
7. Beltrao, P., Albanèse, V., Kenner, L. R., Swaney, D. L., Burlingame, A., Villén, J., Lim, W. A., Fraser, J. S., Frydman, J., and Krogan, N. J. (2012) Systematic functional prioritization of protein posttranslational modifications. *Cell* **150**, 413-425
8. Helbig, A. O., Rosati, S., Pijnappel, P. W., van Breukelen, B., Timmers, M. H., Mohammed, S., Slijper, M., and Heck, A. J. (2010) Perturbation of the yeast N-acetyltransferase NatB induces elevation of protein phosphorylation levels. *BMC Genomics* **11**, 685
9. Winter, D. L., Hart-Smith, G., and Wilkins, M. R. (2018) Characterization of protein methyltransferases Rkm1, Rkm4, Efm4, Efm7, Set5 and Hmt1 reveals extensive post-translational modification. *J. Mol. Biol.* **430**, 102-118
10. Jaiswal, D., Turniansky, R., Moresco, J. J., Ikram, S., Ramaprasad, G., Akinwole, A., Wolf, J., Yates, J. R., and Green, E. M. (2020) Function of the MYND domain and C-terminal region in regulating the subcellular localization and catalytic activity of the SMYD family lysine methyltransferase Set5. *Mol. Cell Biol.* **40**, e00341-19
11. Bodenmiller, B., Wanka, S., Kraft, C., Urban, J., Campbell, D., Pedrioli, P. G., Gerrits, B., Picotti, P., Lam, H., and Vitek, O. (2010) Phosphoproteomic analysis reveals interconnected system-wide responses to perturbations of kinases and phosphatases in yeast. *Sci. Signal* **3**, rs4
12. Swaney, D. L., Beltrao, P., Starita, L., Guo, A., Rush, J., Fields, S., Krogan, N. J., and Villén, J. (2013) Global analysis of phosphorylation and ubiquitylation cross-talk in protein degradation. *Nat. Methods* **10**, 676-682
13. Huber, A., Bodenmiller, B., Uotila, A., Stahl, M., Wanka, S., Gerrits, B., Aebersold, R., and Loewith, R. (2009) Characterization of the rapamycin-sensitive phosphoproteome reveals that Sch9 is a central coordinator of protein synthesis. *Gene. Dev.* **23**, 1929-1943
14. Chen, S.-h., Albuquerque, C. P., Liang, J., Suhandynata, R. T., and Zhou, H. (2010) A proteome-wide analysis of kinase-substrate network in the DNA damage response. *J. Biol. Chem.* **285**, 12803-12812
15. Smolka, M. B., Albuquerque, C. P., Chen, S.-h., and Zhou, H. (2007) Proteome-wide identification of in vivo targets of DNA damage checkpoint kinases. *P. Natl. Acad. Sci. U.S.A.* **104**, 10364-10369
16. Li, X., Gerber, S. A., Rudner, A. D., Beausoleil, S. A., Haas, W., Villén, J., Elias, J. E., and Gygi, S. P. (2007) Large-scale phosphorylation analysis of α-factor-arrested Saccharomyces cerevisiae. *J. Proteome Res.* **6**, 1190-1197
17. Gnad, F., de Godoy, L. M., Cox, J., Neuhauser, N., Ren, S., Olsen, J. V., and Mann, M. (2009) High-accuracy identification and bioinformatic analysis of in vivo protein phosphorylation sites in yeast. *Proteomics* **9**, 4642-4652
18. Soulard, A., Cremonesi, A., Moes, S., Schütz, F., Jenö, P., and Hall, M. N. (2010) The rapamycin-sensitive phosphoproteome reveals that TOR controls protein kinase A toward some but not all substrates. *Mol. Biol. Cell* **21**, 3475-3486
19. Henriksen, P., Wagner, S. A., Weinert, B. T., Sharma, S., Bačinskaja, G., Rehman, M., Juffer, A. H., Walther, T. C., Lisby, M., and Choudhary, C. (2012) Proteome-wide analysis of lysine acetylation suggests its broad regulatory scope in Saccharomyces cerevisiae. *Mol. Cell Proteomics* **11**, 1510-1522
20. Weinert, B. T., Iesmantavicius, V., Moustafa, T., Schölz, C., Wagner, S. A., Magnes, C., Zechner, R., and Choudhary, C. (2014) Acetylation dynamics and stoichiometry in Saccharomyces cerevisiae. *Mol. Syst. Biol.* **10**, 833
21. Tong, Z., Kim, M.-S., Pandey, A., and Espenshade, P. J. (2014) Identification of candidate substrates for the Golgi Tul1 E3 ligase using quantitative diGly proteomics in yeast. *Mol. Cell Proteomics* **13**, 2871-2882
